# Structural Basis of Human Helicase DDX21 in RNA Binding, Unwinding, and Antiviral Signal Activation

**DOI:** 10.1002/advs.202000532

**Published:** 2020-06-08

**Authors:** Zijun Chen, Zhengyang Li, Xiaojian Hu, Feiyan Xie, Siyun Kuang, Bowen Zhan, Wenqing Gao, Xiangjun Chen, Siqi Gao, Yang Li, Yongming Wang, Feng Qian, Chen Ding, Jianhua Gan, Chaoneng Ji, Xue‐Wei Xu, Zheng Zhou, Jinqing Huang, Housheng Hansen He, Jixi Li

**Affiliations:** ^1^ State Key Laboratory of Genetic Engineering Department of Neurology School of Life Sciences and Huashan Hospital Collaborative Innovation Center of Genetics and Development Engineering Research Center of Gene Technology of MOE Shanghai Engineering Research Center of Industrial Microorganisms Fudan University Shanghai 200438 China; ^2^ Department of Neurology Huashan Hospital Fudan University Shanghai 200040 China; ^3^ State Key Laboratory of Genetic Engineering School of Life Sciences Fudan University Shanghai 200438 China; ^4^ Key Laboratory of Marine Ecosystem Dynamics Ministry of Natural Resources & Second Institute of Oceanography Ministry of Natural Resources Hangzhou 310012 China; ^5^ China Novartis Institutes for Biomedical Research Co. Ltd Shanghai 201203 China; ^6^ Department of Chemistry The Hong Kong University of Science and Technology Hong Kong China; ^7^ Department of Medical Biophysics University of Toronto, and Princess Margaret Cancer Center University Health Network Toronto M5G 1L7, Ontario Canada

**Keywords:** ATPases, crystal structures, DDX21, RNA helicases, viral protein NS1

## Abstract

RNA helicase DDX21 plays vital roles in ribosomal RNA biogenesis, transcription, and the regulation of host innate immunity during virus infection. How DDX21 recognizes and unwinds RNA and how DDX21 interacts with virus remain poorly understood. Here, crystal structures of human DDX21 determined in three distinct states are reported, including the apo‐state, the AMPPNP plus single‐stranded RNA (ssRNA) bound pre‐hydrolysis state, and the ADP‐bound post‐hydrolysis state, revealing an open to closed conformational change upon RNA binding and unwinding. The core of the RNA unwinding machinery of DDX21 includes one wedge helix, one sensor motif V and the DEVD box, which links the binding pockets of ATP and ssRNA. The mutant D339H/E340G dramatically increases RNA binding activity. Moreover, Hill coefficient analysis reveals that DDX21 unwinds double‐stranded RNA (dsRNA) in a cooperative manner. Besides, the nonstructural (NS1) protein of influenza A inhibits the ATPase and unwinding activity of DDX21 via small RNAs, which cooperatively assemble with DDX21 and NS1. The structures illustrate the dynamic process of ATP hydrolysis and RNA unwinding for RNA helicases, and the RNA modulated interaction between NS1 and DDX21 generates a fresh perspective toward the virus–host interface. It would benefit in developing therapeutics to combat the influenza virus infection.

## Graphical abstract

RNA helicase DDX21 is associated with many nuclear and nucleolar events, and regulation of host innate immunity. Herein, a set of four crystal structures of human DDX21 are reported, revealing the conformational rearrangements during the RNA unwinding cycle. Furthermore, the discovery of small RNAs modulating the interaction between DDX21 and viral protein NS1 generates a fresh perspective toward the virus–host interface.

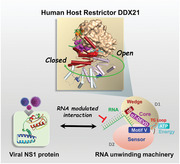

## 1 Introduction

DExD/H‐box RNA helicases unwind RNA structures or dissociate RNA‐protein complexes in cellular processes that require modulation of RNA structures.^[^ ^1^ ^]^ They are vital for the regulation of all aspects of the RNA life cycle, including pre‐mRNA splicing, mRNA export, RNA editing, storage and decay, ribosome biogenesis, and transcription,^[^ ^2^ ^]^ as well as participating in the regulation of protein translation modification,^[^ ^3^ ^]^ cellular signaling pathways,^[^ ^4^ ^]^ and protein localization.^[^ ^5^ ^]^ Furthermore, many DExD‐box helicases exhibit essential functions in innate immunity,^[^ ^6^ ^]^ as well as associated with multiple kinds of cancers,^[^ ^7^ ^]^ making the helicases not only attractive therapeutic targets but also potential biomarkers for cancer diagnosis, prognosis, and therapeutics.

**Figure 1:**
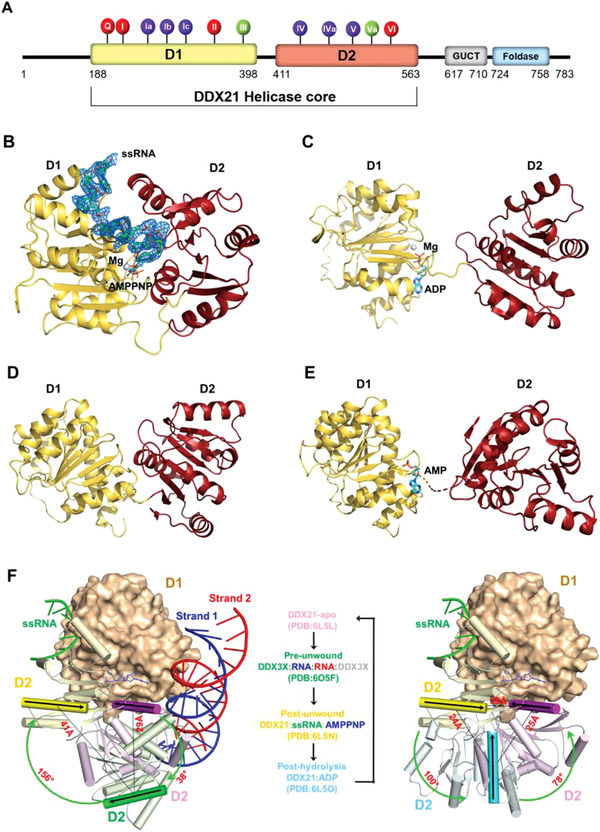
The overall structure of DDX21 helicase core in different unwinding states. A) Domain structure of human DDX21 and conserved sequence motifs of the helicase core. The DDX21 helicase core (D1D2 core, residue 188‐563) was used for structural studies. The 12 highly conserved DDX sequence motifs were colored for their primary functions: red, ATP binding; blue, RNA binding; and green, inter‐domain interaction. See also Figure S1 (Supporting Information). B) Crystal structure of the D1D2 core (yellow/red) bound with ssRNA and AMPPNP‐Mg^2+^. DDX21 is shown in cartoon model, whereas ssRNA is in a stick model with an electron density map contoured to 3.0 *σ* at the *F*_o_–*F*_c_ map. C) Crystal structure of the D1D2 core (yellow/red) bound with ADP‐Mg^2+^. D) Crystal structure of the D1D2 core (yellow/red) in its apo‐state. E) Crystal structure of the D1D2 core (yellow/red) bound with AMP. The disordered part (residues Ile400 – Ile409) connecting the two domains is indicated by a broken line. F) Conformational comparison between the DDX21‐apo (in pink, PDB: 6L5L), DDX3X‐dsRNA (in green, PDB: 6O5F; and only one DDX3X molecule is shown for clarity), DDX21‐ssRNA‐AMPPNP (in yellow, PDB: 6L5N), and DDX21‐ADP (in cyan, PDB: 6L5O) structures. The left panel shows the conformational difference between the apo state, pre‐unwound state and post‐unwound state. The right panel shows the conformational difference between the post‐wound state, post‐hydrolysis and apo state, combining into a four‐step unwinding cycle elucidated in the middle panel. The structures were aligned based on their D1 domains. The D1 of the DDX21‐ssRNA‐AMPPNP structure is illustrated as a molecular surface, and those of other structures are not shown for clarity. The D2 of all four structures are shown as cartoon models (helices as cylinders, strands as arrows, and loops as tubes). The orientation of D2, relative to the fixed D1 at each state, is indicated with a black arrow on the same *α*10 helix in D2 and a color gradient for emphasis. See also Figure 7 and Figure S3 (Supporting Information).

RNA helicase DDX21 contains all signature motifs of the DExD‐box helicase family and three atypical FRGQR repeats and one PRGQR motif (**Figure** **1**A).^[^ ^8^ ^]^ DDX21 is a multifunctional enzyme that has RNA unwinding activity, ATPase activity, and RNA foldase or guanine (G)‐quadruplexes unwinding activity (Figure 1A).^[^ ^9^ ^]^ DDX21 binds various RNAs, such as rRNA, small nucleolar RNA (snoRNA), 7SK small nuclear RNA (snRNA) and at lower extent mRNA, therefore associating with many crucial nuclear and nucleolar events, including ribosomal RNA biogenesis and ribosomal DNA damage,^[^ ^10^ ^]^ unwinding of R‐loops,^[^ ^11^ ^]^ and RNA G‐quadruplexes,^[^ ^12^ ^]^ as well as sensing the transcriptional status of RNA polymerase Pol I.^[^ ^13^ ^]^

DDX21 plays emerging roles in the viral replication and host innate immune response to virus infection, acting as a Toll‐like receptor 3 (TLR3)‐independent viral dsRNA cosensor.^[^ ^14^ ^]^ In this process, DDX21 serves as a bridge between DDX1 and DHX36 and transduces the signal to the adaptor TRIF together with DHX36, to activate the NF‐kB pathway and type I IFN responses to both short and long poly I:C, influenza A virus and reovirus.^[^ ^14^ ^]^ DDX21 also triggers the expression of a damage‐associated molecular pattern (DAMP) protein S100A9, which induces pro‐inflammatory cytokines production and apoptosis through DDX21‐TRIF‐S100A9‐TLR4‐MyD88 signaling network.^[^ ^15^ ^]^ Furthermore, DDX21 directly binds to the REV of HIV and enhances REV oligomerization, promoting the HIV REV‐dependent RNA export to the cytosol.^[^ ^16^ ^]^ Additionally, DDX21 affects ribosomal reinitiation of Bornavirus transcript X via interaction with the 5′‐UTR of X/P mRNA,^[^ ^17^ ^]^ stimulates the innate immune response, reinforcing IFN responses and exerting anti‐Hantaviral effects.^[^ ^18^ ^]^ DDX21 can also act as a host restriction regulator to block influenza A virus replication by direct binding to viral polymerase basic protein 1 (PB1) and inhibiting polymerase assembly, resulting in reduced viral RNA and protein synthesis. Nevertheless, in later stages of infection, this effect is counteracted by the viral NS1 protein, which inhibits the synthesis of interferons.^[^ ^19^ ^]^

Here, we solved the crystal structures of DDX21 in three different states, including the apo‐state, the ADP‐bound free post‐hydrolysis state, and the AMPPNP‐ssRNA bound post‐unwound state. The results provided a comprehensive understanding of the unwinding mechanism, involving utilization of ATP to unwind RNA structures or dissociate RNA‐protein complex in an unwinding cycle, with dramatic conformational changes. Most importantly, we elucidated the RNA‐mediated interaction between DDX21 and viral NS1, and generated an unconventional perspective toward the virus–host interaction.

## 2 Results

### 2.1 Overall Structures of DDX21 in Different States: From “Open” to “Closed” Conformation

**Figure 2:**
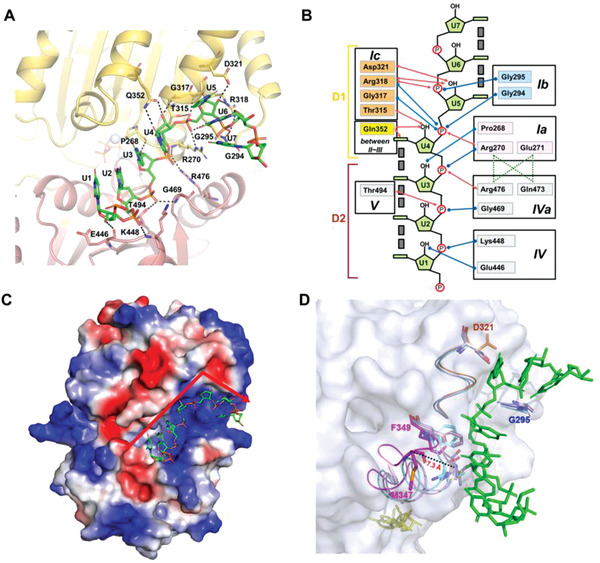
RNA binding and bending in DDX21‐ssRNA‐AMPPNP complex. A) Details of the RNA binding site in DDX21‐RNA‐AMPPNP complex structure. B) Schematic representation of the RNA binding site. Blue and red arrows indicated interactions through the main and the side chains of different residues, respectively. Gray boxes indicate base stacking. C) The electrostatic potential surface of the DDX21‐RNA‐AMPPNP complex. ssRNA is shown in the stick model. The red arrow shows the bent orientation of RNA around the motif Ic. Blue: positive charge; Red: negative charge. D) Comparison of the RNA binding pocket in three DDX21 structures with the surface representation of DDX21‐apo (gray). The four resides from the three motifs that had steric clashes with ssRNA (green) in apo‐state (light blue), AMP‐bound state (light pink) and ADP‐bound state (cyan) were shown as stick models, with the “DF loop” (D321‐F349; magenta), motif Ic (orange), motif Ib (GG; blue), and AMPPNP (yellow). The “DF loop” shifted 7.3 Å between DDX21‐apo and DDX21‐ssRNA‐AMPPNP, measured with the M347 residue.

DDX21 is composed of three domains, including the N‐terminal domain, the middle D1 and D2 domain, and the C‐terminal foldase domain (Figure 1A). To better understand the RNA recognition and unwinding mechanism of DDX21, we determined the crystal structure of human DDX21 helicase core (residue 188–563), encompassing the conserved RecA‐like helicase core domains D1 and D2 in complex with AMPPNP and a 15‐mer poly(U) ssRNA at 2.24 Å resolution (Figure 1B), which represents a post‐unwound or pre‐hydrolysis state in the unwinding cycle. Also, we solved the crystal structures of DDX21 in two other unwound states, the post‐hydrolysis or post‐release state in complex with ADP (Figure 1C), and the apo‐state without any cofactor or RNA (Figure 1D). Moreover, crystal structure of DDX21 with an ADP molecule was solved at 2.7 Å; however, one internal loop was disordered (residues Ile400 – Ile409), and the ADP molecule for crystallization was spontaneously hydrolyzed into AMP (Figure 1E). Further SDS‐PAGE analysis of the DDX21‐AMP crystals indicated that the missing loop was degraded (Figure S1D, Supporting Information). Therefore, we deleted the linker loop partially (GKKTQ; residue 401‐405) and generated a new construct (named as a del‐loop mutant), which helped us obtain a 1.8 Å resolution crystal structure of DDX21‐ADP and another DDX21‐apo structure with 3.1 Å resolution. The inter‐domain interactions between D1 and D2 domains in these two structures help them keep their specific conformations with the retained loop region (Figure S1A–C, Supporting Information). The DDX21‐AMPPNP‐ssRNA ternary complex structure contained two molecules in the asymmetric unit, named as A chain and B chain, respectively, which bound to a poly(U) mRNA mimic, a non‐hydrolyzable nucleotide AMPPNP, and one Mg^2+^ ion. The complex structure revealed a canonical DEAD box RNA helicase core closure process, which brought the N‐terminal ATPase and C‐terminal helicase in close vicinity to create the functional ATPase site and the nucleotide‐binding cleft. Seven bases of the RNA molecule positioned across the top of the cleft, while the N‐terminal D1 domain induced an apparent sharp bend and broke the base stacking of the RNA backbone at the 3′‐end between U4 and U5 (**Figure** **2**A,B). The binding mode and structural basis for unwinding mechanism were consistent with other DEAD‐box RNA helicases (Figure S2, Supporting Information), representative as Vasa^[^ ^20^ ^]^ and DDX19.^[^ ^21^ ^]^

Comparison of the structures of DDX21 involved in the unwinding cycle revealed that the two conserved domains (D1 and D2) of helicase core unambiguously showed an “open” to “closed” conformational change between the apo‐state and post‐unwound state (Figure 1F). Additionally, the ADP‐bound and ssRNA post‐hydrolysis states were precisely in an open‐closed transition state between them (Figure 1F). From the apo‐ to post‐unwound state, D2 domain not only shifted ≈29 Å but also rotated by ≈180°; from the post‐unwound to post‐hydrolysis state, D2 shifted ≈24 Å and rotated by ≈100°; and from the post‐hydrolysis back to the apo‐state, D2 shifted ≈25 Å and rotated by ≈78° (Figure 1F). An entire unwinding cycle by DExD box helicase also includes the pre‐unwound state. However, a structure of DDX21 in complex with dsRNA for this state is not available yet. Previously, two crystal structures for the pre‐unwound state were reported: the Mss116p‐dsRNA structure (PDB: 4DB2),^[^ ^22^ ^]^ and DDX3X‐dsRNA structure (PDB: 6O5F).^[^ ^23^ ^]^ The reported Mss116p‐dsRNA structure only contains the D2 domain, while the DDX3X‐dsRNA structure has an intact D1D2 core. More importantly, a comparison of the relative positions of the bound nucleotides in our closed‐state DDX21‐AMPPNP‐ssRNA structure and the two helicase‐dsRNA structures, revealed that the phosphate groups of nucleotides (U2‐U5) of U15‐ssRNA bound to D1D2 core in the post‐unwound state structure of DDX21, occupied similar positions to the four bound nucleotides (N20‐N23) of RNA strand 1 in the DDX3X D1D2‐dsRNA structure, while they were farther away from the corresponding nucleotides (N4‐N7) in the Mss116p D2‐dsRNA structure (Figure S3, Supporting Information). The ssRNA in the DDX21‐AMPPNP‐ssRNA (PDB: 6L5N) corresponds to part of the strand 1 in the DDX3X‐dsRNA structure (PDB: 6O5F), and they are recognized by both D1 and D2 in the two structures (Figure S3, Supporting Information). Thus, the DDX3X‐dsRNA structure was used to represent the DDX21‐dsRNA complex at the pre‐unwound state. As shown in Figure 1F, from the apo‐ to pre‐unwound state, the D2 domain not only shifted ≈29 Å but also rotated by ≈38°; from the pre‐unwound to post‐unwound state, the D2 shifted ≈41 Å and rotated by ≈156°.

### 2.2 Binding of ssRNA to DDX21

To reveal how DDX21 binds and unwinds RNA, the detailed protein‐RNA interactions were analyzed (Figure 2A). The two domains (D1 and D2) of DDX21 clasped ssRNA, enfolding it within an interaction network that was dominated by polar contacts (Figure 2A). In brief, the interacting residues were highly conserved among the DEAD‐box proteins except for Glu446 and Lys448 within the motif IV, and Gln352 that resided between motif II and motif III (Figure S4, Supporting Information). DDX21 formed eleven hydrogen bonds with the phosphate groups of the RNA backbone and recognized six 2’‐OH groups of RNA backbone, which thereby discriminated against DNA substrates (Figure 2A,B). Uniquely, Gln352 recognized both the base moieties and 2’‐OH group of U4, which was not found in other DEAD‐box proteins (Figure 2A,B). In the seven visible bases of RNA, the 5’‐ends of the RNA (U1‐U2) interacted with the C‐terminal D2 domain including motif IV, motif IVa and motif V. The 3’‐ends of the RNA (U4‐U6) interacted with the N‐terminal D1 domain including motif Ia, motif Ib (or GG motif) and motif Ic, while U7 was free of binding to DDX21. The middle U3 interacted with both D1 and D2 domains through motif Ia and motif Iva, respectively (Figure 2A,B). For the overall structure of bound RNA, the U1‐U2‐U3‐U4 bases and the U5‐U6‐U7 bases were individually stacked, but the U4 and U5 bases were not stacked with each other. Thus the RNA was bent between U4 and U5, and the bent region was located near the DDX21 motif Ic within the conserved *α*6 helix, the so‐called “wedge” helix (Figure 2A,B). This “wedge” helix disrupts base pairs by bending one of the strands, when a duplex is bound.^[^ ^20^ ^]^ The arrangement of (U1‐U4):(U5‐U7) from 5’‐end to 3’‐end explained clearly the preference of the kind of RNA substrate containing a single‐stranded tail, attached to the 5’ terminus of one strand of the duplex for unwinding, as previously reported.^[^ ^9^ ^]^ The 5’‐ssRNA tail could be caught by DDX21 and accommodated at the U1‐U3 position in the crystal structure, thus unwinding the duplex more efficiently.

The kink of ssRNA greatly benefited from the nearby positive electrostatic surface (Figure 2C), composed of three arginine residues (Arg270, Arg318, and Arg476) locating around the kink joint between U4 and U5 (Figure 2A,B). Arg318 formed multiple interactions with RNA, not only with the U4 phosphate but also with the U5 phosphate and the U5 2’‐OH group. Besides, U4 and U5 were extensively recognized by residues Gly294, Gly295, Thr315, Gly317, Asp321, and Gln352 (Figure 2A,B). Furthermore, many inter‐domain interactions between D1 and D2 were formed around the RNA binding sites (Figure S2, Supporting Information), leading to the helicase core closure. Residues Arg270 and Arg476 stacked to form inter‐domain interactions with Gln473 and Glu271, respectively, whereas Gln473 interacted with Glu271. Thus Arg270 and Arg476 were fixed at their positions to interact with U4 and U3 phosphate by their side chains (Figure 2B).

**Figure 3:**
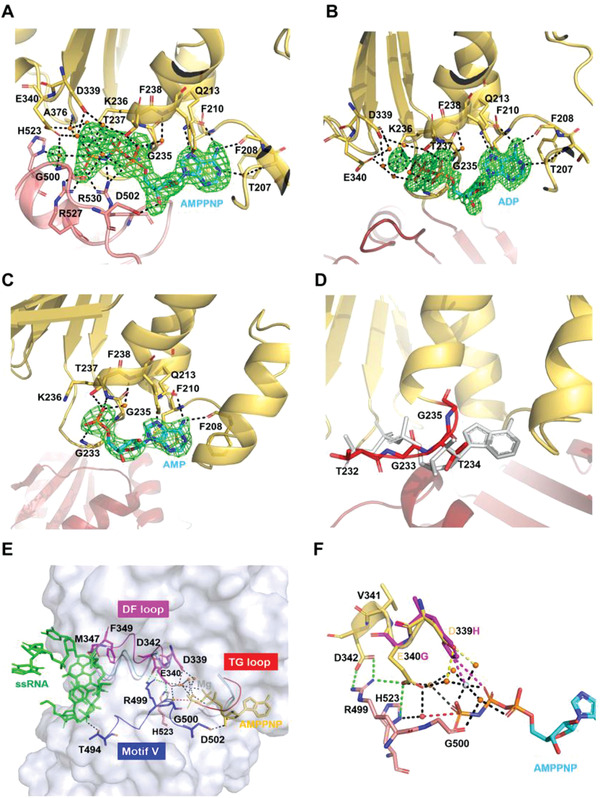
The ATPase sites of DDX21. A) Representation of the ATPase sites in DDX21‐RNA‐AMPPNP complex, with the electron density map (*F*_o_ – *F*_c_) for AMPPNP‐Mg^2+^ and the presumptive catalytic water rendered at 3.0 *σ*. The broken red line connects the probable attacking water and the *γ*‐phosphorus atom. Arg204 is omitted for clarity. B) The ADP binding sites in DDX21‐ADP complex, with the electron density map (*F*_o_ – *F*_c_) for ADP‐Mg^2+^ rendered at 3.0 *σ*. Arg204 is omitted for clarity. C) The AMP binding sites in DDX21‐AMP complex, with the electron density map (*F*_o_ – *F*_c_) for AMP rendered at 3.0 *σ*. D) The potential ATP binding sites in the DDX21‐apo structure. The “TG loop” is colored in red. The modeled AMPPNP is colored in gray. E) Stereo view of the link between ATP binding pocket and RNA binding pocket, with the surface representation of DDX21‐ssRNA‐AMPPNP (gray). In the DDX21‐ssRNA‐AMPPNP structure, the “DF loop” is indicated in magenta, motif V (and Va) in blue, the “TG loop” in red, AMPPNP in yellow and RNA in green. In the DDX21‐apo structure, the “DF loop” is colored in light blue, and the “TG loop” is colored in red. Both of the “DF loop” and “TG loop” were colored in cyan in the DDX21‐ADP structure and light pink in the DDX21‐AMP structure. Green broken lines show the inter‐domain interactions. F) Superimposition of D339H and E340G mutants. D339H forms a new salt bridge (purple) with ATP phosphate and new cooperative bond (purple) with Mg^2+^ ion by the His substitution, while E340G mutation lacks the inter‐domain interactions with Arg499 and His523. Yellow broken lines show the interaction of Asp339 with Mg^2+^, mediated by water. Green broken lines show the inter‐domain interactions of Glu340 with Arg499 and His523 and inter‐domain interaction of Asp342 with Arg499. The broken red line shows the interaction between the presumptive attacking water and the *γ*‐phosphorus atom.

Different from the observed “open to closed” conformational changes between the two conserved domains, a “reverse” transition from relatively closed to open RNA binding pocket was found in the three unwound states (Figure 2D). When DDX21 was in the apo‐state, the RNA binding pocket was closed tightly (Figure 2D). Three motifs in the D1 domain sterically clashed with the modeled ssRNA molecule, including residue Gly295 from motif Ib, residue Asp321 from motif Ic, and more significantly, residues Met347 and Phe349 adjacent to Gln352 which interacted with U4 in the RNA bound structure (Figure 2D). There was a dramatic conformational change within a loop, spanning from DEVD box within motif II to the residue Phe349 (named as “DF loop”; Figures 2D and **3**E), exhibiting a consecutive increasing distance to ssRNA that shifted ≈7.3 Å from apo‐state to ssRNA bound post‐unwound state (Figure 2D). The “DF loop” and Asp321 in ADP‐bound structure had clashes with modeled ssRNA (Figure 2D), which was consistent with its post‐hydrolysis state, otherwise the bound RNA would be unstable and released. Only when ATP binding favored closure of the inter‐domain cleft that led to the formation of the extended RNA‐binding surface (Figure 2C) and “DF loop” was far away from bound RNA, the RNA binding pocket was exposed and the RNA kink was stabilized (Figure 2D).

### 2.3 Linkage between the Binding Pockets of ATP and ssRNA

The ATP‐binding pocket was formed between the closed D1 and D2 inter‐domain cleft (Figure 3A). The presumptive attacking water for ATP hydrolysis was close to the *γ*‐phosphorus atom of AMPPNP and interacted with the side chains of Glu340 (motif II) in the D1 domain, His523 (motif VI) and the amide group of Gly500 (motif Va) in D2 domain. Furthermore, AMPPNP interacted substantially with residues from motif Q, motif I, motif II and motif III within the D1 domain, and residues from motif Va and motif VI within the D2 domain (Figure 3A). Mainly, motif V and adjacent motif Va not only interacted with RNA and AMPPNP, but were also involved in several inter‐domain interactions, which rendered this region to be a sensor between RNA and ATP binding sites that were critical for RNA unwinding activities (Figure 3E). Besides, the AMPPNP interacting residues Glu340 (motif II), Ser375, Thr377 (motif III), and His523 (motif VI) participated in the inter‐domain interactions (Figure S1, Supporting Information). Therefore, the bound ATP, attacking water molecule, and RNA interacted extensively with the pockets created by the two conserved domains in the closed form.

The motif I had a dramatic conformational change by comparison among different states (Figure 3A–E). A short loop (TGTG, named as “TG loop”) within the motif I had severe steric hindrance with the modeled AMPPNP in DDX21 apo‐structure; thus the ATP‐binding pocket was in the closed conformation (Figure 3D). While in AMPPNP, ADP, or AMP bound states, the “TG loop” was open, and therefore accommodated ligand binding (Figure 3E). More importantly, only in the AMPPNP‐bound form, the residues Asp339 and Glu340 within the DEVD box bound to the *γ*‐phosphorus atom through Mg^2+^ ion and water molecules, and incorporated with multiple inter‐domain interactions. The interactions, in turn, established more contacts with the presumptive attacking water and stabilized the interactions with *γ*‐phosphorus (Figure 3E). Consequently, the conformational change of DEVD box motif transduced to the other end of previously described “DF loop” and led to the opening of RNA binding pocket, as residues Met347 and Phe349 would not block the RNA binding anymore (Figures 2D and 3E).

**Figure 4:**
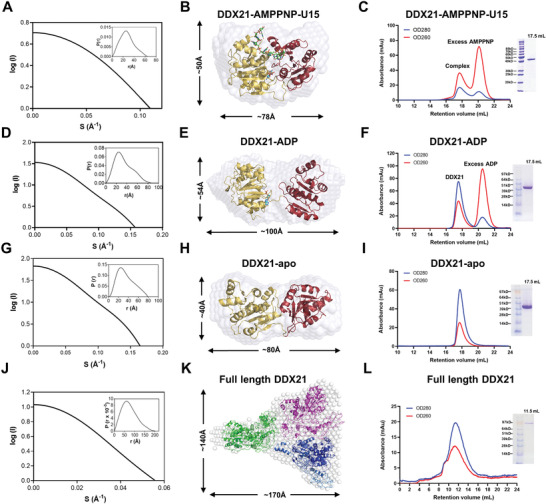
SAXS models and gel‐filtration profiles of full‐length DDX21 and the helicase core. A) The experimental scattering curve and the distance distribution function curve (inset) for DDX21 helicase core (188‐563) with AMPPNP and U15 ssRNA. B) Crystal structure of DDX21‐AMPPNP‐ssRNA (PDB: 6L5N) was fitted into the ab initio envelope obtained from SAXS. C) Gel filtration profile and the SDS‐PAGE of DDX21 helicase core (188‐563) with AMPPNP and U15 ssRNA. The helicase core and U15 ssRNA plus AMPPNP were coeluted out at the retention volume 17.6 mL from the superdex200 10/300 column (GE healthcare, Boston, USA), and excess AMPPNP was eluted out lately. D) The experimental scattering curve and the distance distribution function curve (inset) for DDX21 helicase core (188‐563) with ADP. E) Crystal structure of DDX21 (del‐loop)‐ADP (PDB: 6L5O) was fitted into the ab initio envelope obtained from SAXS. F) Gel filtration profile and the SDS‐PAGE of DDX21 helicase core (188‐563) with ADP. The helicase core with ADP was eluted out at the retention volume 17.5 mL from the superdex200 10/300 column (GE healthcare, Boston, USA), and excess ADP was eluted out lately. G) The experimental scattering curve and the distance distribution function curve (inset) for DDX21 helicase core (188‐563). H) Crystal structure of DDX21 (del‐loop)‐apo (PDB: 6L5L) was fitted into the ab initio envelope obtained from SAXS. I) Gel filtration profile and the SDS‐PAGE of DDX21 helicase core (188‐563). The helicase core was eluted out at the retention volume 17.6 mL from the superdex200 10/300 column (GE healthcare, Boston, USA), corresponding with a molecular weight around 30 kD, indicating the possible monomer status in solution. J) The experimental scattering curve and the distance distribution function curve (inset) for full‐length DDX21. (K) The modeled structure of full‐length DDX21 was fitted into the SAXS ab initio envelope with three DDX21 molecules. The modeled DDX21 structure was obtained using the Phyre2 web portal (http://www.sbg.bio.ic.ac.uk/~phyre/). L) Gel filtration profile and the SDS‐PAGE of full‐length DDX21 (1‐783). DDX21 was eluted out at the retention volume 11.6 mL from the superdex200 10/300 column (GE healthcare, Boston, USA), corresponding with a molecular weight around 340 kD, indicating the possible trimer or tetramer status in solution.

To further clarify the conformational change of DDX21 upon binding with RNA, the different solution states were investigated by using the small‐angle X‐ray scattering (SAXS) method.^[^ ^24^ ^]^ The SAXS patterns of DDX21‐AMPPNP‐U15, DDX21‐ADP, and DDX21‐apo were recorded to generate the final composite scattering curves (**Figure** **4**A,D,G). The maximum dimensions (Dmax) from the distance distribution function *p*(*r*) for the three states were 66.58, 88.47, and 77.3 Å, respectively. When superimposing the crystal structures into their corresponding ab initio shape envelopes, they fitted very well (Figure 4B,E,H). Their gel filtration profiles were also shown in Figure 4C,F,I, respectively.

### 2.4 Coupling of ssRNA Binding and RNA Unwinding

**Figure 5:**
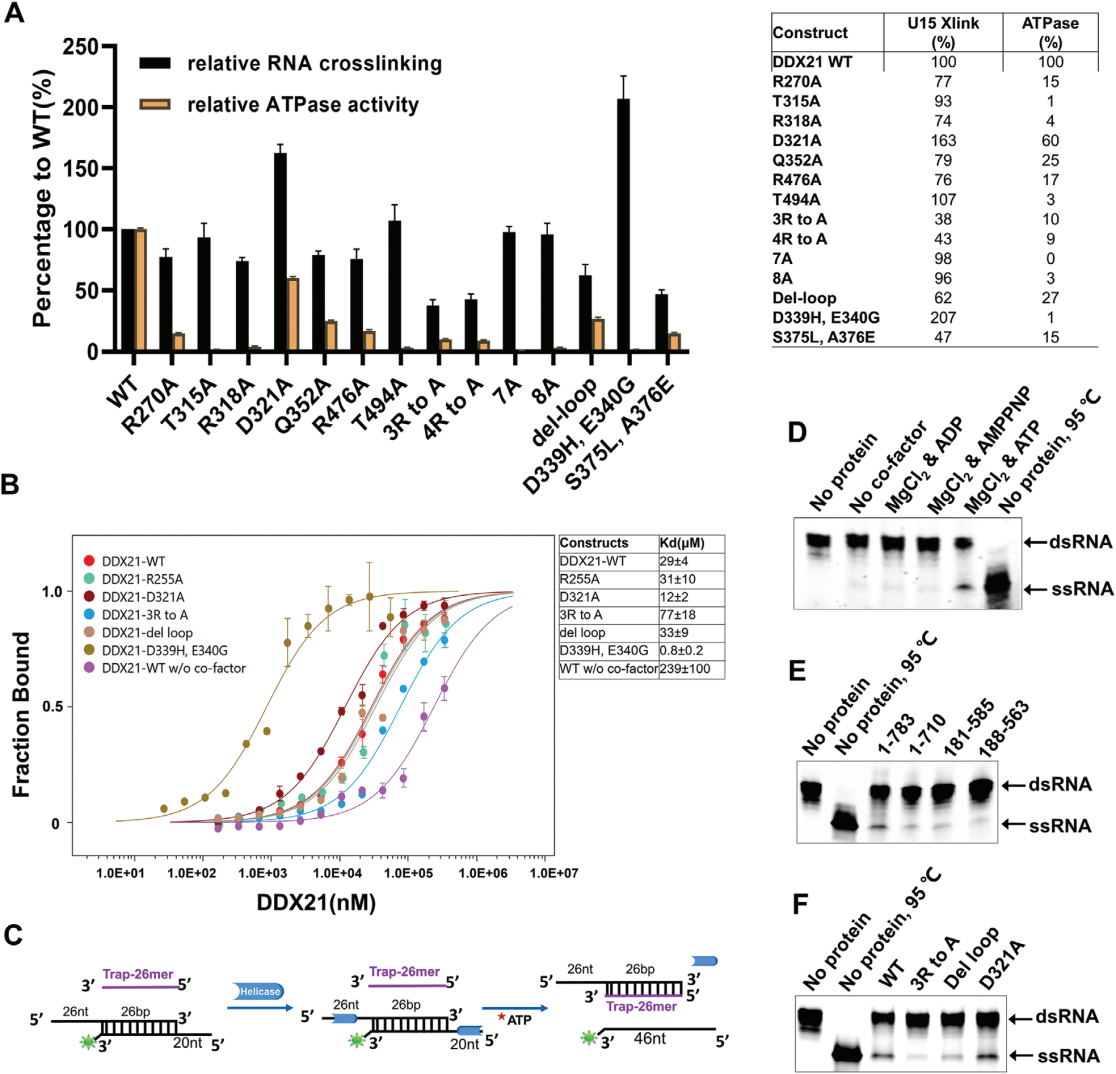
Mutational analysis of key residues involved in ATP hydrolysis and RNA binding. A) Histogram (left panel) and detailed values (right panel) of the RNA‐crosslinking and RNA‐stimulated ATPase activities, which are shown as percentages of the wide‐type DDX21 activity. All presented data represent means ±S.D. of three independent determinations. B) The U15 binding affinities of WT and mutant DDX21. Error bars represent the standard error between three replicate experiments. C) Schematic diagram of the unwinding process with 5’‐tailed RNA duplex and trap RNA. D) Full‐length DDX21 unwinds RNA duplex with or without different cofactors. The final products of unwinding reaction were separated by a 4–20% gradient Novex TBE Gel (Thermo Fisher Scientific, Waltham, USA). DDX21 unwound 5′‐tailed RNA duplex effectively with ATP and MgCl_2_, while very weak bands of ssRNA were still observed from other reaction pools without ATP. E) RNA unwinding with full length and different truncated forms of DDX21 by using a 4–20% gradient Novex TBE Gel (Thermo Fisher Scientific, Waltham, USA). The unwinding reactions were proceeded with 2 × 10^−3^ m ATP and 2 × 10^−3^ m MgCl_2_ by using 2 × 10^−6^ m DDX21 full‐length or truncations, 0.2 × 10^−6^ m 5′‐tailed RNA duplex, and 0.8 × 10^−6^ m unlabeled trap RNA. F) RNA unwinding with the full‐length WT and mutant DDX21, by using a 4–20% gradient Novex TBE Gel (Thermo Fisher Scientific, Waltham, USA), and the same reaction conditions described above in (E).

The DDX21‐ssRNA structure revealed that all the thirteen conserved motifs were involved in the ATP binding, RNA binding, and inter‐domain interaction, except the Vb motif (Figure S4, Supporting Information). Mutational analyses were performed to understand the roles of those conserved residues. First, RNA binding affinity was examined by UV‐induced RNA‐crosslinking (**Figure** **5**A), MST, and SEC coelution. Briefly, full‐length DDX21 bound to U15 RNA, and unwound a 5’‐tailed RNA duplex substrate in an ATP‐dependent manner (Figure 5B–D), which was in line with previous studies.^[^ ^9^ ^]^ Interestingly, a very low ATP‐independent unwinding activity for DDX21 was also observed (Figure 5D), similar to the inspections in the recent study.^[^ ^25^ ^]^ The helicase core of DDX21 maintained the RNA binding affinity but exhibited a decreased ATPase activity and a much weaker unwinding activity when compared with full‐length DDX21 (Figure 5E and Figure S5A–C, Supporting Information). Therefore, the accessory domains flanking the DEAD helicase core might function synergistically during RNA unwinding and remodeling. Different polynucleotides including poly(U) and poly(C), synthetic 15‐mer poly(U) and 10‐mer poly(U) (named as U15 and U10, respectively) were used to study ATPase activity (Figure S5A–C, Supporting Information), which was best stimulated by poly(U), revealing discrepancy to the previous reports on a distinct preference of poly(C).^[^ ^8^ ^]^ U15 had a higher stimulation affinity with DDX21 and formed a more stable complex with DDX21 than that of U10, which was evidenced by MST assay (Figure S5D, Supporting Information) and SEC coelution analysis (Figure S5E,F, Supporting Information). However, U10 could not form a similar stable complex in SEC coelution experiment, although only 7‐mer of poly(U) was visible in the electron density of crystal structure.

Second, 16 mutants of DDX21 were used to investigate the effects on RNA‐crosslinking and ATPase activity (Figure 5A and Figure S6, Supporting Information). They included Arg270Ala, Thr315Ala, Arg318Ala, Asp321Ala, Gln352Ala, Arg476Ala, Thr494Ala, “4R to A” mutant (namely Arg255Ala/Arg270Ala/Arg318Ala/Arg476Ala), “8A” mutant (the eight residues were all mutated to alanine), Asp339His and Glu340Gly (from the conserved DExD motif), Ser375Leu and Ala376Glu (from conserved SAT motif), and the del‐loop mutant.

The results showed that single point mutation Arg270Ala, Arg318Ala, Gln352Ala, or Arg476Ala led to the apparent loss of the RNA‐crosslinking activity, but not so effective as illustrated in the previous study with Vasa protein.^[^ ^20^ ^]^ Nonetheless, the “3R to A” mutant (Arg270Ala, Arg318Ala, and Arg476Ala) or “4R to A” mutant remarkably reduced the RNA binding affinity by more than half. In contrast, the “8A” mutant and “7A” mutant (excluding R255A) retained comparable RNA‐crosslinking activity with WT DDX21. This might result from the Asp321Ala mutation, which probably enhanced the RNA‐crosslinking by the elimination of the electrostatic repulsion between the Asp321 side chain and the RNA phosphates. The “4R to A” and “8A” mutants had similar results as “3R to A” and “7A” mutants, respectively, indicating that Arg255Ala did not involve with the RNA binding and ATPase activity. Similarly, Thr315Ala and Thr494Ala abolished the ATPase activity but negligibly affected RNA‐crosslinking activity (Figure 5A and Figure S6, Supporting Information).

The mutants Asp339His, Glu340Gly, Ser375Leu, and Ala376Glu significantly reduced the ATPase activity of DDX21 (Figure S6C,D, Supporting Information). Furthermore, the Asp339His/Glu340Gly mutant exhibited barely detectable ATPase activity, while mutation Ser375Leu/Ala376Glu still retained some degree of ATPase activity (Figure S6C,D, Supporting Information). In contrast, the Asp339His/Glu340Gly had the highest RNA‐crosslinking activity, while Ser375Leu/Ala376Glu kept only about half of the activity (Figure 5A,B). The increase of RNA‐crosslinking of Asp339His/Glu340Gly might be caused by the formation of the new salt bridge with ATP phosphate and a new cooperative bond with Mg^2+^ ion by the His substitution (Figure 3F). A similar increase of ATP binding for DEAD mutants was observed in DEAD‐box helicase elF‐4A.^[^ ^26^ ^]^ However, in contrast to the increase of ATP binding by Asp339His mutation, the Glu340Gly mutation lost the inter‐domain interaction with Arg499 and His523 (Figure 3F). Since His523 was the critical residue to coordinate ATP hydrolysis through direct interaction with the presumptive attacking water, the ATP hydrolysis activity would be lost and, thus the unwinding activity was blocked due to the lack of the necessary “power stroke” upon ATP hydrolysis for completing the catalytic cycle. In fact, according to the previous reports, the two mutants should be both utterly defective in unwinding.^[^ ^11^ ^]^ Interestingly, despite retaining more than half of the RNA‐crosslinking activity, the ATPase activity of del‐loop mutation was more affected as the unwinding activity (Figure 5F). This result suggested that the loop region between the D1 and D2 domains might play a role in both ATPase and unwinding activities, probably through influencing the inter‐domain interaction. Finally, the “3R to A” mutant lost most of the unwinding activity, whereas the Asp321Ala mutant had comparable unwinding activity (Figure 5F).

### 2.5 DDX21 Unwinds dsRNA Cooperatively

Our SAXS and gel filtration data showed that full‐length DDX21 might be a trimer in solution (Figure 4J–L). To test the impact of oligomerization on the catalytic activities of DDX21, we measured ATP‐dependent RNA unwinding under pre‐steady state conditions following the protocol previously described for Ded1p,^[^ ^27^ ^]^ with enzyme excess resulted in a sigmoidal functional binding isotherm for full‐length DDX21. The Hill coefficient of *H* = 2.7 ± 0.3 indicated cooperation between at least three DDX21 protomers (Figure S7A–C, Supporting Information), in agreement with our SAXS and gel filtration data. DDX21 D1D2 core showed significantly lower unwinding activity than full‐length DDX21 at comparable concentrations. The Hill coefficient of *H* = 1.3 ± 0.3 indicated a monomeric state of DDX21‐core for unwinding (Figure S7D, Supporting Information). These data provided evidence that the oligomerization of DDX21 is critical for optimal helicase activity, and that the accessory domains flanking the D1D2 core are vital for the unwinding oligomer.

We then measured functional binding isotherms for RNA‐dependent ATPase activity. Functional affinities of both full‐length DDX21 and DDX21‐core for the ATPase activity were similar to those for the unwinding activity. The Hill coefficients were *H* = 3.3 ± 0.4 for full‐length DDX21 (Figure S7E, Supporting Information), and *H* = 1.2 ± 0.2 for D1‐D2 core (Figure S7F, Supporting Information). Together, the results of Hill cooperativity analysis provide direct evidence for RNA duplex unwinding by full‐length DDX21 cooperatively.

### 2.6 Cooperative Assembly of DDX21, NS1, and Small RNAs

Previous studies have shown that non‐structural (NS1) protein of influenza A virus could overcome host restriction on RNA polymerase assembly and the synthesis of viral RNA and protein by binding to DDX21 and displacing PB1.^[^ ^19^ ^]^ To better understand the mechanism of NS1 interaction with DDX21, we coexpressed full‐length His‐tagged DDX21 and Flag‐tagged NS1 in HEK293T cells, and copurified them by two‐step affinity chromatography. Gel filtration profile revealed a 1:1 stoichiometry ratio in the DDX21‐NS1 complex, which was coupled with high apparent nucleic acid contents, inferred from the higher absorbance of OD260 over OD280 (Figure S8A, Supporting Information). We then extracted the nucleic acid by phenol/chloroform, followed by ethanol/Na acetate precipitation. The OD260/OD280 ratio of extracted nucleic acid was close to 2.0, which indicated that it was most likely RNA but not DNA. The extracted RNA size was close to 17 nt (data not shown). The subsequent analysis by Agilent 2100 Bioanalyzer confirmed that these were small RNA fragments with a length between 10–20 nt (Figure S8B and Table S1, Supporting Information). The SAXS and gel filtration data showed that DDX21 helicase core was a monomer (Estimated M.W. = 49.8 kD), and the full‐length DDX21 might be a trimer in solution (Figure 4G–L). However, the trials of SAXS experiments of the purified DDX21‐NS1 complex indicated that it might be polymerized in solution with a massive size (Dmax*smin>*π*; data not shown). This result established that DDX21, NS1, and small RNAs cooperatively assembled into a complex, and NS1 possibly interacted with DDX21 via RNA.

### 2.7 Inhibition of DDX21 ATPase and Unwinding Activity by NS1

To investigate whether NS1 affects the ATPase activity and unwinding activity of DDX21, we performed in vitro assays. NS1 inhibited DDX21 ATPase activity in a dose‐dependent manner, more efficiently at lower RNA concentration (Figure S8D, Supporting Information). When the stimulated RNA was decreased from 5 to 0.2 × 10^−6^ m, NS1 exhibited nearly complete inhibition of DDX21 ATPase activity at a molar ratio of 4:1 (Figure S8D, Supporting Information). Similarly, the truncated DDX21 helicase core (DDX21‐ΔNC) showed a dose‐dependent ATPase inhibition by a truncated NS1 (1‐215) protein. The same dose‐dependent manner was observed with different concentrations of U15 RNA (Figure S8E, Supporting Information), which indicated that DDX21 helicase core was involved in the interaction with NS1. Furthermore, the inhibition was dramatically relieved when an RNA‐binding defective DDX21 mutant (3R to A) was introduced instead of WT DDX21. On the contrary, when Asp321Ala mutant with increased RNA binding affinity was used, the inhibition was enhanced (Figure S8F, Supporting Information).

RNA helicase assays were performed by using different concentrations of NS1 (Figure S8C, Supporting Information). When the titration ratio between NS1 and DDX21 was increased from 1:1 to 4:1, a complete inhibition effect for all doses was observed. However, when titrated NS1 down to four‐hundredth of DDX21, the dose‐dependent inhibition occurred (Figure S8C, Supporting Information). More interestingly, the complete inhibition started at a molar ratio of 20:1 (DDX21:NS1), which clearly showed that the inhibition was precisely through RNA. Since we used a one‐tenth molar ratio of RNA duplex substrate to DDX21, the final ratio between RNA duplex and NS1 was 1:2, which is in accordance with previous reports on forming of NS1A homo‐dimer by binding to double‐stranded RNA.^[^ ^28^ ^]^ Taken together, these data provided evidence that NS1‐dependent inhibition on DDX21 was highly correlated to the NS1 ability for binding with RNA substrates, either by titrating away the active RNA reaction pools or by directly inhibiting DDX21 activity in a DDX21‐RNA‐NS1 assembly. Considering the DDX21‐NS1 cooperative complex assembly with small RNAs (Figure S8A, Supporting Information), the existence of a ternary complex of DDX21‐RNA‐NS1 was highly predictable and that NS1 might interact with DDX21 in an RNA‐mediated manner (Figure 7).

### 2.8 Counteraction of NS1 on DDX21 Antiviral Activity in Infected Cells is via RNA

**Figure 6:**
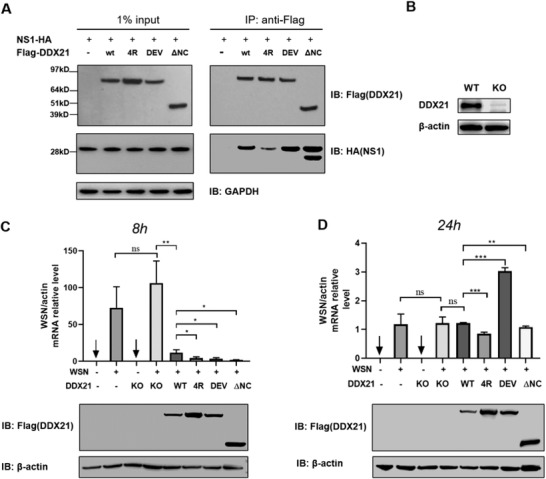
NS1 counteracts DDX21 activity in the cells. A) Coimmunoprecipitation of NS1 with WT and mutated DDX21 from cotransfected HEK293T cells. B) Immunoblotting analysis for endogenous DDX21 in HEK293T cells. C,D) Viral RNA synthesis level determination with quantitative real‐time RT‐PCR. qRT‐PCR analysis of viral RNA in wild‐type and DDX21 knock‐out HEK293T cells that transfected with indicated plasmids at C) 8 h post‐infection and D) 24 h post‐infection with WSN. *P < 0.05, **P < 0.01, ***P < 0.001, and ns, not significant (Student's *t*‐test, error bars, s.e.m., *n* = 3).

Next, to investigate how NS1 counteracted DDX21 in vivo, Flag‐tagged WT and mutant DDX21, or truncated DDX21 helicase core (ΔNC) together with HA‐tagged NS1 were cotransfected into HEK293T cells. As shown in **Figure** **6**A, the RNA‐binding defective mutant of DDX21 (4R to A) was associated with less of NS1 while the DDX21 DEV mutant (Asp339His, Glu340Gly) could pull‐down more NS1 protein than WT DDX21. Additionally, the DDX21 helicase core pulled‐down much more NS1, demonstrating that the helicase core was not only involved but also played essential roles in the interaction with NS1, which was consistent with the in vitro results (Figure S8E,F, Supporting Information).

We further examined the effects of WT and mutant DDX21 on the inhibition of viral RNA synthesis in a DDX21 knock‐out HEK293T cell line infected with influenza virus WSN (Figure 6B). WT DDX21 could effectively rescue the inhibition of virus replication at the early time of infection, while both “4R to A” and DEV mutants showed more substantial inhibition effects than WT DDX21 (Figure 6C). At a later time of infection, with the accumulation of virus NS1 protein, the inhibition of DDX21 was relieved in WT and mutants (Figure 6D). Interestingly, while “4R to A” still showed a more potent inhibition, DEV mutant demonstrated a total reverse inhibition by NS1 (Figure 6D), which was in line with its most potent binding ability of DEV mutant to RNA (Figure 5A,B). These data showed that DDX21 and NS1 interacted with each other through RNA, since the counteracting abilities from virus NS1 correlated positively with the RNA binding ability of DDX21.

## 3 Discussion

**Figure 7:**
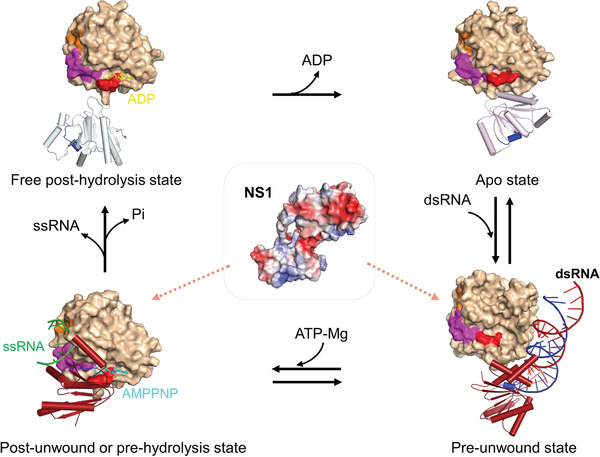
A proposed model for DDX21 unwinding cycle associated with NS1. A four‐step unwinding cycle represented by the apo‐ (PDB: 6L5L, this work), pre‐unwound (PDB: 6O5F), post‐unwound (PDB: 6L5N, this work), and post‐hydrolysis (PDB: 6L5O, this work) states. Full‐length NS1 might bind to DDX21 through RNA in its pre‐unwound state or post‐unwound state. The D1 of all the structures are illustrated as a molecular surface (light orange), and the D2 domains are shown as cartoon models (helices as cylinders, strands as arrows, and loops as tubes). On the D1 surface, the orange color represents motif Ic (the “wedge” helix); the magenta color represents the “DF loop” spanning from DEVD box within motif II to the residue Phe349; the red color represents the “TG loop” within the motif I; the blue color in D2 domain represents the motif V (the “sensor”). DDX3X‐dsRNA structure (PDB: 6O5F) is used here to represent DDX21 pre‐unwound state and only one DDX3X molecule is shown for clarity. The corresponding motifs in the DDX3X structure are shown in the same colors as for DDX21. All these motifs within both D1 and D2 have significant conformational changes during the unwinding cycle.

DDX21 is associated with many crucial nuclear and nucleolar events, as well as coordinating rRNA processing in cancer^[^ ^16^ ^]^ and regulation of host innate immunity.^[^ ^29^ ^]^ However, the nature of the regulatory mechanism of DDX21 remained unclear. Here, we have shown that the DDX21 helicase core adopted an open conformation in its apo‐state, and the success of crystallization either without the linker loop or del‐loop constructs indicated the vast flexibility of the open conformation in solution. A closed conformation of the DDX21 helicase core was well‐defined in its post‐unwound complex with ATP and ssRNA. Interestingly, the ADP‐bound helicase core exhibited an “intermediate state” of the closure of D1 and D2 domains (Figure 1F). We also uncovered intriguing conformational changes of the “TG loop” and “DF loop” that located in the ATP binding pocket and RNA binding pocket, respectively, and how they linked with each other through DEVD‐box. First, the apo‐D1D2 core bound dsRNA in an ATP independent manner. Then, ATP binding led to dsRNA unwinding. In this process, ATP binding triggered the “TG loop” conformational change and led to the following conformational change of DEVD box upon core closure, which then induced the third wave of conformational change to “DF loop” and led to the expose of ssRNA binding pocket. And the “wedge” helix disrupted base pairs by bending one of the strands of dsRNA. In this process, the “sensor” motif V and Va (commonly combined and named as motif V in some literature) tethered RNA and ATP, and formed extra inter‐domain interaction with DEVD‐box, which stimulated the conformational change of DEVD‐box. Next, after ATP hydrolysis, the interaction network around the *γ*‐phosphorus collapsed, and the “DF” loop returned to similar conformation as for apo‐state; thus, the bound ssRNA was destabilized and subsequently released. Finally, further release of ADP brought the D1D2 core back to the apo‐state, and allowed for the next round of the catalytic cycle. The whole process illustrates how the motifs mentioned above in D1 and D2 cooperatively assemble as efficient accessories for an unwinding machine (**Figure** **7**).

The helicases DDX3X and Ded1p cooperatively unwind dsRNA.^[^ ^23^, ^27^, ^30^ ^]^ Hill cooperativity analysis indicated that both full‐length Ded1p and full‐length DDX3X function as a trimer for unwinding. The participation of a third molecule in full‐length DDX3X optimizes the unwinding efficiency, whereas the D1D2 core of DDX3X exhibits a two‐molecule cooperativity.^[^ ^23^ ^]^ For ATP hydrolysis, full‐length Ded1p and DDX3X D1D2 core exhibit a two‐molecule cooperativity, suggesting that the third DDX does not hydrolyze ATP.^[^ ^23^, ^27^, ^30^ ^]^ Our Hill cooperativity analysis showed that full‐length DDX21 exhibited a three‐molecule cooperativity for dsRNA unwinding, whereas the D1D2 core showed a monomeric state with significantly lower unwinding activity than full‐length DDX21 (Figure S7A–D, Supporting Information). For the ATPase activity, Hill cooperativity analysis showed that full‐length DDX21 exhibited a three‐molecule cooperativity. In contrast, the D1D2 core still showed a monomeric state (Figure S7E,F, Supporting Information). Taken together, these data provide direct evidence that the oligomerization of DDX21 is critical for optimal helicase activity, but the detailed mechanism of action in dsRNA recognition and unwinding might be different with that of DDX3X/Ded1p subfamily. Both Ded1p and DDX3X have a low complexity region (LCR) of about 60 amino acid residues in their C‐terminus, which is responsible for the recruitment of the third protomer that plays the RNA‐loading role and does not hydrolyze ATP.^[^ ^23^, ^27^, ^30^ ^]^ Sequence analysis showed that DDX21 has a LCR‐like sequence in its C‐terminal tails (residues 693‐768), but with lower identity (≈22.4% identity to Ded1p). This LCR‐like region of DDX21 contains the foldase domain, which is rich in glycine and arginine residues and contains three FRGQR repeats followed by a final PRGQR sequence, a unique pattern that does not appear in other human proteins.^[^ ^12^ ^]^ Interestingly, our gel filtration data supported that DDX21 del‐C (1‐710) was highly polymerized and might be a pentamer in solution, while DDX21 del‐NC (188‐710) should be a dimer (data not shown); this indicated that the N‐terminus and/or C‐terminus of DDX21 (including the foldase domain) should be critical for the trimer formation, while C‐terminus of DDX21 might also be important to maintain the protein stability. Moreover, the GUCT domain (residues 617‐710), while not critical for the trimer formation, might be part of the protein‐protein interface in the dimer, probably through its hydrophobic surface.^[^ ^31^ ^]^ However, the exact mechanism of how the GUCT domain and the foldase domain affect the ATPase and unwinding activities of DDX21 waits to be further elucidated.

The NS1 of influenza A virus functions in regulating viral replication, host innate/adaptive immune responses, and cellular signaling pathways.^[^ ^32^ ^]^ DDX21 binds to the NS1 N‐terminal RNA‐binding domain (RBD), and the association is RNase A‐resistant, assuming that NS1 binds DDX21 in an RNA‐independent manner.^[^ ^19^ ^]^ However, as RNase A is specific to ssRNA and only cleaves 3’‐ end of unpaired U and C, it could not cleave polyA or RNA duplex.^[^ ^33^ ^]^ Of particular importance, RNAs in an RNA‐protein complex are always liable to be protected by the bound proteins and the efficiency of cleavage by RNase is often limited to buffer condition and the reaction time.^[^ ^34^ ^]^ NS1 RBD can interact with polyG or polyA of mRNAs, stem/bulge regions of dsRNAs, and other dsRNAs in a sequence‐independent manner,^[^ ^35^ ^]^ while its C‐terminal effector domain (ED) is involved only in protein–protein interactions.^[^ ^36^ ^]^ So, if DDX21 binds to NS1 through its RBD domain, it is reasonable that RNA plays roles in the protein–protein interactions. Additional evidence might come from the in vitro reconstitution of the ternary complex of DDX21‐RNA‐NS1 (Figure S9, Supporting Information). Although the full‐length DDX21‐NS1 complex was not stable enough under the physiological salt concentration with or without RNA, probably due to the intrinsic instability of the proteins, our copurification results established the cooperative assembly of DDX21, NS1, and small RNAs. The DDX21‐RNA‐NS1 axis could be a potentially useful antiviral drug target‐directed against influenza A virus, since vaccines are not guaranteed to be effective.

## 4 Conclusions

In summary, here we reported multiple sets of crystal structures of RNA helicase DDX21 either in ligand‐free (DDX21‐apo) or in complex with AMP (DDX21‐AMP), ADP (DDX21‐ADP), or a nonhydrolyzable ATP analog (AMPPNP) and a 15‐nucleotide poly(U) ssRNA (DDX21‐AMPPNP‐U15), corresponding to different unwinding states (except for DDX21‐AMP). We elucidated the conformational rearrangements upon the ATP and ssRNA binding during the dsRNA unwinding cycle, and described an RNA unwinding machinery including one wedge helix, one sensor motif V, and the DEVD box. Hill coefficient analysis revealed that DDX21 unwound dsRNA in a three‐molecule cooperative manner. Besides, it was found that the nonstructural (NS1) protein of influenza A inhibited both ATP‐hydrolyzing and RNA‐unwinding activity of DDX21 via small RNAs, which played a crucial role in forming the DDX21‐RNA‐NS1 ternary complex. Our findings here would help to further understand the virus–host interface and thus provide a rational strategy in developing therapeutics to influenza virus infection.

## 5 Experimental Section

##### Cloning, Protein Expression, and Purification

The human DDX21 (NP_004719.2 in GenBank) and viral protein NS1 (P03495 in Uniprot) were synthesized with a codon‐optimization strategy by Genewiz (Suzhou, China). The WT and mutants of DDX21 were expressed in pET28 (Novagen, Darmstadt, Germany) vector with an N‐terminal His‐sumo tag in BL21‐CodonPlus (DE3)‐RIL cells. Briefly, cells were suspended in buffer A (20 × 10^−3^ m Tris‐HCl, pH 7.5, 1 m NaCl, 5% glycerol, 0.2 × 10^−3^ m DTT), supplemented with complete EDTA‐free protease inhibitor cocktail tablets (Roche, Basel, Switzerland), and lysed by a high‐pressure homogenizer. DDX21 proteins were then purified by Ni‐NTA affinity chromatography, and the His‐sumo tag was removed by ULP1 cleavage followed with additional Ni‐NTA affinity chromatography. The target proteins were then applied to a Superdex 200 gel filtration column pre‐equilibrated in buffer B (20 × 10^−3^ m Tris‐HCl, pH 7.5, 100 × 10^−3^ m NaCl, 1 × 10^−3^ m TCEP). The WT and helicase core of DDX21 was finally concentrated to around 30 mg mL^−1^. For full‐length (1‐783) and del‐C (1‐710) DDX21 proteins, the final SEC buffer included 500 × 10^−3^ m NaCl and 200 × 10^−3^ m NaCl, respectively. Full‐length NS1 (1‐237) and NS1 (1‐215) were expressed with an N‐terminal His‐tag plasmid and purified as previously reported.^[^ ^3^ ^]^ NS1 RBD domain (1‐72) was fused with an N‐terminal GST tag, followed with a TEV cleavage site, and purified using GST affinity chromatography. Buffer B was used in SEC for all NS1 proteins, except for full‐length NS1, for which the same buffer containing 500 × 10^−3^ m NaCl and 5% glycerol was used.

##### Crystallization and Data Collection

All crystals were grown by the sitting‐drop vapor diffusion method. Purified DDX21 (188‐563) was diluted to 6–8 mg mL^−1^ and incubated with a 15‐mer poly(U) RNA at a protein to RNA ratio of 1:1.1 in 20 × 10^−3^ m Tris‐HCl (pH 7.5) buffer containing 100 × 10^−3^ m NaCl, 1 × 10^−3^ m TCEP, 1.5 × 10^−3^ m AMPPNP, and 3 × 10^−3^ m MgCl_2_. The DDX21‐ssRNA crystals were grown at 20 °C by mixing 1 µL of the above sample with 1 µL of reservoir solution, comprised of 7% MPD and 0.1 m Bicine, pH 8.5. Crystals of DDX21‐AMP were grown at 4 °C, and 20 mg mL^−1^ of DDX21 (188‐563) protein supplemented with 5 × 10^−3^ m ADP and 10 × 10^−3^ m MgCl_2_ was mixed with an equal volume of reservoir solution (19% PEG3350, 0.18 m ammonium citrate, 40 × 10^−3^ m myo‐inositol) with crystal seeds. Crystal appeared within two weeks. For the DDX21 del‐loop‐ADP crystals, 20 mg mL^−1^ of DDX21 (188‐563, del‐loop) protein supplemented with 5 × 10^−3^ m ADP and 10 × 10^−3^ m MgCl_2_ was mixed with an equal volume of reservoir solution containing 20% pEG3350, 0.2 m Magnesium acetate and crystals were grown at 20 °C. The del‐loop apo‐DDX21 crystals were grown at 20 °C by mixing 20 mg mL^−1^ of DDX21 with an equal volume of reservoir solution (20% PEG3350 and 0.2 m Na_2_HPO_4_).

Diffraction data were collected with crystals flash‐frozen in crystallization buffer supplemented with 30–35% v/v glycerol. Integration, scaling, and merging of the diffraction data were performed using the XDS program package^[^ ^38^ ^]^ and HKL2000.^[^ ^39^ ^]^

##### Structure Determination

For the structural determination of the DDX21‐AMP complex, the helicase core of Hera from Thermus thermophiles (PDB: 4KBF)^[^ ^40^ ^]^ was used as a searching model using PHASER.^[^ ^41^ ^]^ Structure refinement was performed with REFMAC5^[^ ^42^ ^]^ and PHENIX.^[^ ^43^ ^]^ Iterative rounds of the model building were performed using COOT.^[^ ^44^ ^]^ The other three structural determinations of the RNA‐bound complex, ADP‐bound complex, and the apo‐DDX21 were solved by using the DDX21‐AMP structure as a search model. The molecular graphics were prepared using PyMol (Schrodinger, New York, USA). The dataset and refinement statistics were summarized in **Table** **1**.

**Table 1:** Data collection and refinement statistics

	DDX21‐AMPPNP‐U15	DDX21‐ADP	DDX21‐AMP	DDX21‐apo
PDB code	6L5N	6L5O	6L5M	6L5L
**Data collection**				
Space group	P2_1_	P2_1_	P2_1_	C2
Unit cell *a*,*b*,*c* (Å)	72.36, 89.23, 79.69	66.46, 43.83, 72.44	36.39, 219.07, 126.39	146.17, 46.63, 58.49
*α*, *β*, *γ* [°]	90 115.6 90	90 115.64 90	90 90.79 90	90 101.16 90
Resolution range (Å)^a)^	40.00–2.24 (2.36–2.24)	43.83–1.80 (1.90–1.80)	40.00–2.70 (2.85–2.70)	36.15–3.10 (3.27–3.10)
Total reflections	276183 (41221)	139159 (19644)	219751 (33408)	26341 (3993)
Unique reflections	43794 (6378)	33164 (4904)	51933 (7930)	6479 (952)
Multiplicity	6.3 (6.5)	4.2 (4.0)	4.2 (4.2)	4.1 (4.2)
Completeness (%)	99.7 (99.9)	94.6 (96.1)	95.8 (99.7)	90.5 (92.1)
*I*/*σ* (I)	11.6 (3.7)	7.9 (2.3)	6.4 (2.5)	9.8 (4.5)
Wilson B‐factor	28.57	13.86	30.36	56.23
*R*_merge_ ^b)^	0.091 (0.449)	0.129 (0.673)	0.209 (0.682)	0.123 (0.325)
*R*_meas_ ^c)^	0.109 (0.536)	0.145 (0.765)	0.239 (0.780)	0.140 (0.370)
*R*_pim_ ^d)^	0.059 (0.29)	0.065 (0.354)	0.113 (0.374)	0.065 (0.172)
*R*_sym_ ^e)^	0.091 (0.449)	0.129 (0.673)	0.209 (0.682)	0.123 (0.325)
CC_1/2_ ^f)^	0.996 (0.853)	0.994 (0.722)	0.976 (0.774)	0.988 (0.930)
**Refinement statistics**				
Resolution (Å)	33.33–2.24 (2.32–2.24)	36.91–1.80 (1.86–1.80)	36.51–2.70 (2.72–2.70)	34.12–3.10 (3.17–3.10)
Reflections used in refinement	43766 (4376)	33156 (3299)	51895 (1038)	6479 (382)
Reflections used for *R*_free_	2106 (190)	1713 (171)	2580 (49)	349 (22)
*R*_work_ ^g)^	0.175 (0.224)	0.175 (0.230)	0.207 (0.258)	0.191 (0.209)
*R*_free_ ^h)^	0.233 (0.289)	0.209 (0.286)	0.257 (0.306)	0.241 (0.268)
No. residues	754	372	1830	368
**RMSD**				
Bond lengths (Å)	0.012	0.007	0.01	0.008
Bond angles (Å)	1.28	0.87	1.13	1.02
Average B‐factor	37.69	20.73	25.7	33.14
Ramachandran favored (%)	98.8	99.19	97.4	96.11
Ramachandran allowed (%)	1.2	0.81	2.1	3.61
Ramachandran outliers (%)	0	0	0.5	0.28
**No. atoms**				
Protein or RNA	6266	2969	14470	2889
Ligands or ions	96	34	115	–
water	158	148	58	–
**B‐factors**				
Protein or RNA	37.96	20.53	25.8	30.25
Ligands or ions	30.66	17.82	21.85	–
Water	31.29	25.32	8.89	–

a) Values in parentheses are for the highest resolution shell

b) *R*_merge_ = ∑|(*I*_i_ −<*I*>) |/∑|*I*|, where *I*_i_ is the intensity of an individual reflection and is the average intensity of that reflection

c) *R*_meas_, redundancy‐independent (multiplicity‐weighted) *R*_merge_

d) *R*_pim_, precision‐indicating (multiplicity‐weighted) *R*_merge_

e) *R*_sym_ = ∑|(*I* −<*I*>) |/∑|*I*|, where I is the observed intensity

f) CC_1/2_ is the correlation coefficient of the mean intensities between two random half‐sets of data

g) *R*_work_ = ∑||*F*_o_| − |*F*_c_||/∑|*F*_o_|, where *F*_o_ and *F*_c_ are the observed and calculated structure factors for reflections, respectively

h) *R*_free_ was calculated as *R*_work_ using the 5% of reflections that were selected randomly and omitted from refinement.

##### Small Angels X‐Ray Scattering (SAXS)

The SAXS data were collected at beamline BL19U2 of the Shanghai Synchrotron Radiation Facility, with a radiation wavelength of 1.03 Å. The protein samples were prepared at concentrations of 1–3 mg mL^−1^ in 20 × 10^−3^ m Tris‐HCl (pH 7.5), 100 × 10^−3^ m NaCl, and 1 × 10^−3^ m TCEP for DDX21 (188‐563) and 20 × 10^−3^ m Tris‐HCl (pH 7.5), 500 × 10^−3^ m NaCl, and 1 × 10^−3^ m TCEP for DDX21 (1‐783). Each blank or sample was measured in triplicate, and the sample measurements were adjusted by subtracting the scattering from buffer alone. Data were analyzed using the software package BioATSAS (https://www.embl‐hamburg.de/biosaxs/). The scattering images were averaged and subtracted from the buffer‐scattering images. Using the indirect Fourier transform method, the Rg was estimated. The distribution function p(r) was calculated from the parameter as the Dmax. The SAXS envelope of full‐length or truncated DDX21 was built by the program GASBOR, as previously reported.^[^ ^37^ ^]^

##### RNA‐Crosslinking

The RNA‐crosslinking assay was carried out by incubating 2 × 10^−6^ m DDX21 with 2 × 10^−6^ m 5’‐Fam labeled 15‐mer poly(U) RNA in 20 × 10^−3^ m Tris‐HCl (pH 7.5) buffer, containing 50 × 10^−3^ m NaCl and 1 × 10^−3^ m TCEP, 5 × 10^−3^ m MgCl_2_ and 2 × 10^−3^ m AMPPNP at room temperature for 30 min. The reaction mixtures were then irradiated with a 254 nm UV lamp for 30 min. After irradiation, samples were subjected to SDS‐PAGE on a 4–12% polyacrylamide gel, which was finally analyzed by a Gel Doc XR imaging system (Bio‐Rad, Hercules, USA).

##### Microscale Thermophoresis (MST)

To perform MST, WT or mutant DDX21 proteins were prepared in MST buffer (50 × 10^−3^ m Tris, pH 7.5, 50 × 10^−3^ m KCl, 2 × 10^−3^ m MgCl_2_, 1 × 10^−3^ m TCEP, 0.01% NP‐40, 0.05 mg mL^−1^ BSA and 2 × 10^−3^ m AMPPNP). Both the labeled RNA and the protein samples were centrifuged at 20 000 × *g* for 10 min to remove aggregates. Binding reactions were prepared in MST buffer to a total volume of 15 µL. Protein samples were serially diluted to obtain a concentration range spanning from ≈0.1 × Kd to ≈10 × Kd, where possible, and mixed with Fam‐labeled 15‐mer or 10‐mer poly(U) ssRNA, which was held constant at a final concentration of 50 × 10^−9^ m. Premium coated capillaries were used for all MST measurements on the Monolith NT. All samples were prepared and measured in triplicates. The resulting signals from thermophoresis were analyzed using the affinity analysis software (NanoTemper Technologies, Munich, Germany) and fitted to the Kd model with the equation

(1) $$\begin{array}{ccc} & & f\left(c_{\mathrm{ligand}}\right)=\mathrm{Unbound}+\left(\mathrm{Bound}-\mathrm{Unbound}\right)\\ & & \;\times \;\frac{\begin{array}{c} c_{\mathrm{ligand}}+c_{\mathrm{target}}+\mathrm{Kd}-\sqrt{{\left(c_{\mathrm{ligand}}+c_{\mathrm{target}}+\mathrm{Kd}\right)}^{2}-4\cdot c_{\mathrm{ligand}}\cdot c_{\mathrm{target}}} \end{array}}{2c_{\mathrm{target}}} \end{array}$$

where *f*(*c*_ligand_) is the Fnorm value with a given protein concentration *c*_ligand_; Unbound is the *F*_norm_ signal of the target alone; Bound is the *F*_norm_ signal of the complex; Kd is the dissociation constant or binding affinity; and *c*_target_ is the final concentration of RNA in the assay.

##### ATPase Assay

ATPase activities of the DDX21 proteins (1 × 10^−6^ m protein in buffer containing 20 × 10^−3^ m Tris‐HCl, pH 7.5, 2 × 10^−3^ m ATP, 5 × 10^−3^ m MgCl_2_, 1 × 10^−3^ m TCEP) were measured using the EnzChek phosphate assay kit (Invitrogen, Carlsbad, USA) at 37 °C. The assay was performed in either the presence or the absence of RNA (0.2 mg mL^−1^ poly(U)/poly(C) RNA (Sigma‐Aldrich, Saint Louis, USA) or 5 × 10^−6^ m 10‐mer/15‐mer poly(U) (Bioneer, Oakland, USA). All data represent means ± S.D. of three independent determinations. NS1 inhibition on DDX21 ATPase activity was performed by adding full‐length NS1 (1‐237) or NS1‐ΔC (1‐215) into the reaction pool with different concentration of U15, and full‐length NS1 (1‐237) was used to block full‐length DDX21 ATPase activity. All samples in this group were kept consistent with 150 × 10^−3^ m NaCl in the above‐mentioned buffer. NS1‐ΔC was used to block the ATPase activity of DDX21‐ΔC (188‐563) WT or mutants. For NS1 inhibition, ATPase activities were normalized to the activity in the absence of NS1. Hill coefficient was measured in the pre‐steady state by using 100 × 10^−9^ m 5′‐tailed RNA duplex to stimulate ATP hydrolysis and titration of DDX21 concentrations. ATPase rates and functional binding parameters were fitted using GraphPad Prism 7.0 (GraphPad software, San Diego, USA) as described.^[^ ^23^, ^27^, ^30^ ^]^

##### RNA Unwinding Assay

The annealed 5’‐tailed RNA duplex substrate^[^ ^8^ ^]^ and unlabeled trap RNA (CGGUACCCGGGGAUCCUCUAGAGUCG) were purchased from HuaGen Biotech (Shanghai, China). FAM fluorescein was added to the 3’‐end of lower strand RNA in the RNA duplex. The unwinding of the duplex was monitored by following the displacement of the labeled strand at 37 °C. The reaction mixture contained 20 × 10^−3^ m Tris‐HCl, pH 7.5, 50 × 10^−3^ m KCl, 1 × 10^−3^ m TCEP, 2 × 10^−3^ m MgCl_2_, 5% glycerol, 2 × 10^−3^ m ATP, and 1 U mL^−1^ RNasin (Thermo Fisher Scientific, Waltham, USA), 2 × 10^−6^ m DDX21, 0.2 × 10^−6^ m RNA duplex, and 0.8 × 10^−6^ m unlabeled trap RNA. After incubation at 37 °C for 60 min, aliquots (10 µL) were mixed with 0.2 µL 5% SDS and 1 µL 10 mg mL^−1^ proteinase K, and then incubated for 20 min at 37 °C. The mixtures were subjected to a TBE native PAGE with a 4–20% polyacrylamide gel. The strands were visualized by ChemiDoc MP Imaging System (Bio‐Rad, Hercules, USA). NS1 inhibition on unwinding was performed by adding full‐length NS1 (1‐237) with DDX21 in different molar ratios. Hill coefficient was measured in the pre‐steady state by using 10 × 10^−9^ m 5’‐tailed RNA duplex substrate and titration of DDX21 concentrations. Furthermore, the strands were visualized by Typhoon FLA 9000 Gel Imaging Scanner (GE healthcare, Boston, USA). Unwinding rates and functional binding parameters were fitted using GraphPad Prism 7.0 (GraphPad software, San Diego, USA) as described.^[^ ^23^, ^27^, ^30^ ^]^

##### Cells

HeLa and HEK293T cell lines were kept in the lab and were cultured in high‐glucose‐containing DMEM supplemented with 10% v/v FBS (Hyclone, Logan, USA). All cells were grown at 37 °C in a 5% CO_2_ incubator (Thermo Fisher Scientific, Waltham, USA). PolyJet (SignaGen, Frederick, USA) or Lipofectamine RNAiMAX (Invitrogen, Carlsbad, USA) was used for the transfection of corresponding plasmids or siRNAs into HeLa cells.

##### Copurification of DDX21‐NS1 from Transfected HEK293T Cells and RNA Sequencing

Full‐length (FL) DDX21 (N‐terminal His tagged) and NS1 (C‐terminal Flag‐tagged) were cotransfected into HEK293T cells and coexpressed at 37 °C for 2 days. The cells were collected and lysed in buffer (20 × 10^−3^ m Tris‐HCl, pH 7.5, 500 × 10^−3^ m NaCl, 0.2 × 10^−3^ m DTT), supplemented with complete EDTA‐free protease inhibitor cocktail tablets (Roche, Basel, Switzerland), and purified by Ni‐NTA affinity chromatography, followed with anti‐flag affinity chromatography. RNA, copurified with the complex, was extracted using phenol/chloroform pH 4.5, followed by ethanol/Na acetate precipitation at −80 °C overnight. RNA pellets were washed with 75% ethanol and dissolved in 50 µL water. The extracted RNA samples were examined with agarose gel and urea polyacrylamide gel, and submitted to Genewiz (Suzhou, China) for small RNA sequencing by Illumina HiSeq. Briefly, RNA sample was quantified and qualified by Agilent 2100 Bioanalyzer and 2 µg RNA sample was used for next‐generation sequencing library preparation according to the manufacturer's protocol (NEBNext Multiplex Small RNA Library Prep Set for Illumina). Small RNA was ligated with 5’‐SR Adaptor and 3’‐SR adaptor for Illumina, and the first‐strand cDNA was synthesized using ProtoScript II Reverse transcriptase and then amplified by PCR for 12 cycles using P5 and P7 primers. The PCR products were recovered and cleaned up using PAGE. The libraries with different indexes were multiplexed and loaded on an Illumina HiSeq instrument according to manufacturer's instructions (Illumina, CA, USA). The sequences were processed and analyzed by Genewiz (Suzhou, China).

##### Coimmunoprecipitation (co‐IP) of DDX21‐NS1 Complex

N‐terminal Flag‐tagged full‐length DDX21 and its mutants or truncated DDX21 helicase core and C‐terminal HA‐tagged full‐length NS1 were cotransfected into HEK293T cells. Cells were collected after 48 h post‐transfection and lysed in a buffer consisting of 25 × 10^−3^ m Tris‐HCl 7.5, 150 × 10^−3^ m NaCl, 5% glycerol, 0.1 × 10^−3^ m DTT supplemented with complete EDTA‐free protease inhibitor cocktail tablets (Roche, Basel, Switzerland). After centrifugation, the cell supernatants were used for co‐IP assay with anti‐Flag beads (Sigma‐Aldrich, Saint Louis, USA) and the elutes from anti‐Flag beads were then loaded to SDS‐PAGE and transferred to a nitrocellulose membrane for sequential immunoblotting analysis with anti‐Flag M2‐HRP or anti‐HA‐HRP antibodies Sigma‐Aldrich, Saint Louis, USA).

##### CRISPR‐Cas9‐Mediated Gene Knock‐Out

For targeting DDX21 with CRISPR/Cas9, an epiCRISPR construct was used.^[^ ^45^ ^]^ The guide RNA sequences (DDX21‐gRNA1: 5′‐ggagcccattgaaaagaaag‐3′; DDX21‐gRNA2: 5′‐cttcactggcagcttcactg‐3′) were designed using the online tool (http://crispr.mit.edu/) that was developed by Feng Zhang lab. 2.5 µg of the epiCRISPR plasmid (with cloned sgRNA) was transfected into HEK293T cells for 48 h, followed by selection with 2 µg mL^−1^ puromycin (Life Technologies, Carlsbad, USA) for 7–10 days. Then, genomic DNA was extracted from cells using QuickExtract DNA Extraction Solution (Lucigen, Middleton, USA) following the manufacturer's protocol. The gRNA targeting sites were PCR‐amplified using Q5 High‐Fidelity DNA polymerase (NEB, Ipswich, USA). The PCR products were purified and were sequenced by Sanger's sequencing, and the genome editing conditions were detected. For analysis of single cell‐derived clones, the cells were disassociated into single cell at day 15 post‐transfection, and seeded in the 96‐well plates with a puromycin‐free medium for 15 days. Individual clones were picked and genotyped. Protein expression was determined by immunoblotting analysis.

##### Quantitative Reverse Transcription‐Polymerase Chain Reaction (qRT‐PCR)

Total RNA was isolated from cells using TRIZOL (Sigma‐Aldrich, Saint Louis, USA), and cDNA synthesis was performed using 1 µg of total RNA with All‐in‐one RT MasterMix (ABM, Richmond, Canada). qRT‐PCR was done with gene‐specific primers. H‐actin: forward, 5’‐ACCAACTGGGACGACATGGAGAAA‐3’ and reverse, 5’‐TAGCACAGCCTGGATAGCAACGTA‐3’; Influenza NA vRNA: forward, 5’‐ATGGATTAGCCATTCAATTCA‐3’ and reverse, 5’‐ACCCACGGATGGGACAA‐3’. For western blot analysis, cells were washed with cold PBS and lysed in a RIPA buffer. Proteins were separated on 10%‐12% polyacrylamide gels and transferred into NC membranes. Membranes were blocked in 5% bovine serum albumin (BSA) and then incubated with primary antibodies, respectively, including anti‐Flag (Proteintech, Rosemont, USA), anti‐DDX21 (Abcam, Cambridge, USA), anti‐influenza virus NS1 (Santa Cruz, Dallas, USA), and anti‐*β*‐Actin (Proteintech, Rosemont, USA). Proteins were then visualized using SuperSignal West Dura Extended Duration Substrate (Thermo Fisher Scientific, Waltham, USA).

##### Statistical Analysis

Each experiment was performed at least three times. All experiment data were analyzed using GraphPad Prism 7.0 (GraphPad software, San Diego, USA) and were presented as mean values ± SD. The statistical significance was performed using unpaired Student's *t*‐test or one‐way/two‐way ANOVA followed by Bonferroni's multiple comparisons test (*: *p* < 0.05; **: *p* < 0.005; ***: *p* < 0.0005).

## Conflict of Interest

The authors declare no conflict of interest.

## Supporting information

Supporting information
