# Supplementary material for: Structural Basis of Human Helicase DDX21 in RNA Binding, Unwinding, and Antiviral Signal Activation
Source: Adv Sci (Weinh). 2020 Jun 8;7(14):2000532. doi: 10.1002/advs.202000532 (PMC7375243; doi:10.1002/advs.202000532)
Supplement: Supplementary file 1 — Supporting information [file ADVS-7-2000532-s001.pdf]

Copyright WILEY-VCH Verlag GmbH & Co. KGaA, 69469 Weinheim, Germany, 2018.

Supporting Information

Structural Basis of Human Helicase DDX21 in RNA Binding, Unwinding, and Antiviral Signal Activation

*Zijun Chen, Zhengyang Li, Xiaojian Hu, Feiyan Xie, Siyun Kuang, Bowen Zhan, Wenqing Gao, Xiangjun Chen, Siqi Gao, Yang Li, Yongming Wang, Feng Qian, Chen Ding, Jianhua Gan, Chaoneng Ji, Xue-Wei Xu, Zheng Zhou, Jinqing Huang, Housheng Hansen He, and Jixi Li**

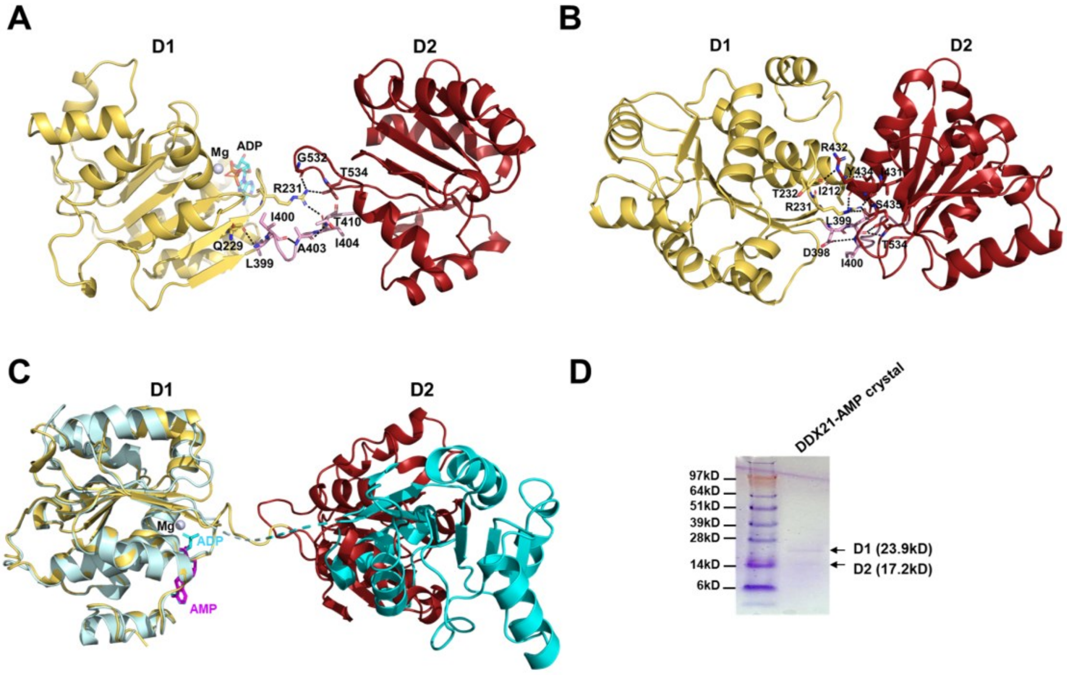

Figure S1. Overall conformation analysis for D1 and D2 domains in DDX21 structures without RNA

(A) Interaction between D1 (yellow), D2 (red) domains and the linker loop (pink) in DDX21-ADP structure (PDB: 6L5O).

(B) Interaction between D1 (yellow), D2 (red) domains and the linker loop (pink) in DDX21-apo structure (PDB: 6L5L).

(C) Superimposition of DDX21-ADP structure (PDB: 6L5O; D1 in yellow and D2 in red) with DDX21-AMP structure (PDB: 6L5M; D1 in pale-cyan and D2 in cyan). The two structures were aligned based on D1.

(D) SDS-PAGE analysis of DDX21-AMP crystals. The crystals were fished out and washed with reservoir buffer for 3 times through a 0.22 μm Costar Spin-X plastic centrifuge tube filters (Corning, Corning, USA), then dissolved into water and analyzed by Coomassie-stained SDS-PAGE. The two bands between 14 kD and 28 kD are corresponding with the D1 and D2 domain respectively in the DDX21-AMP structure, indicated the degradation of the linker loop during crystallization.

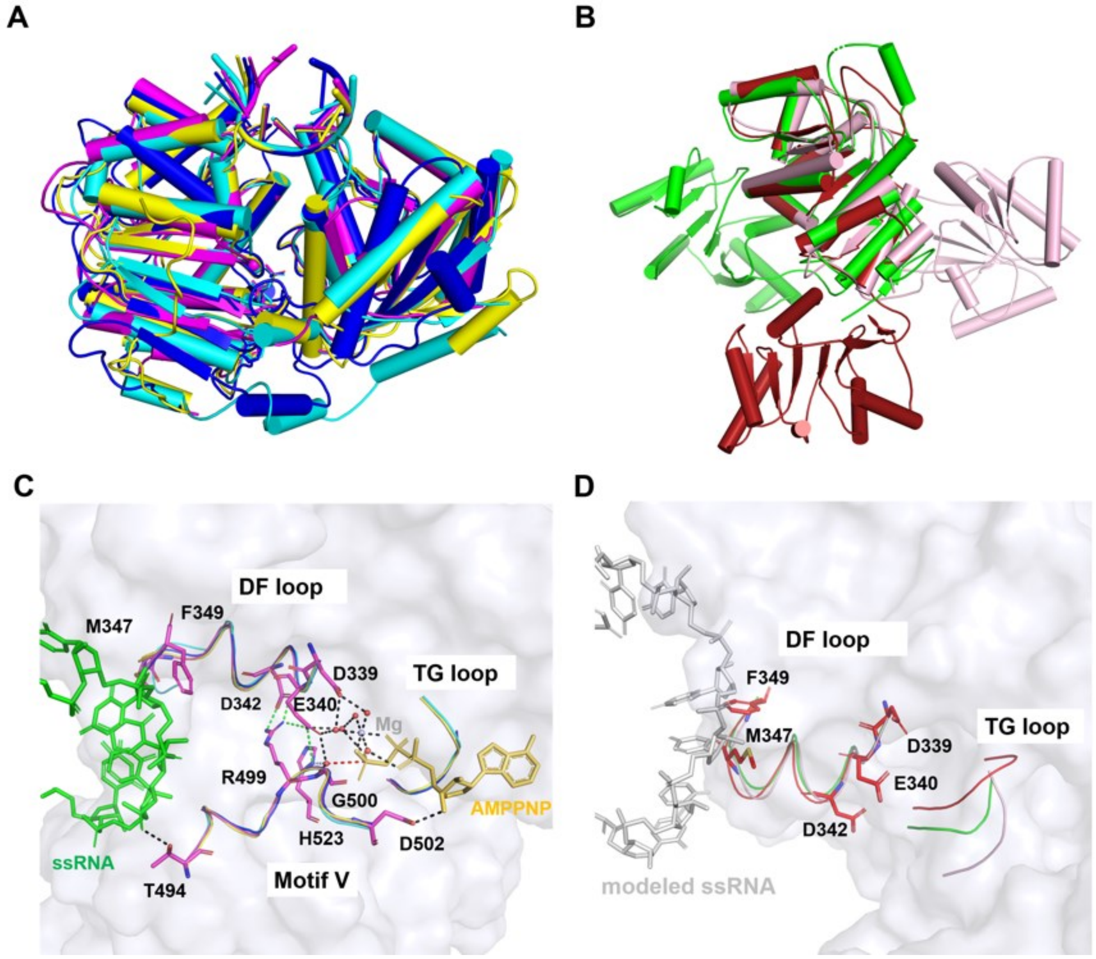

Figure S2. Structure analysis of DDX21-AMPPNP-ssRNA

(A) Superimposition of DDX21-AMPPNP-ssRNA structure (PDB: 6L5N; magenta) with structures of Vasa-AMPPNP-ssRNA (PDB: 2DB3; blue), DDX19-AMPPNP-ssRNA (PDB: 3GOH; cyan) and DDX48-AMPPNP-ssRNA (PDB: 2HYI; yellow). Root mean square deviations (RMSDs) among DDX21 and the other three structures were 0.714 Å, 0.857 Å, and 0.839 Å, respectively.

(B) Superimposition of DDX21-apo structure (PDB: 6L5L; red) with DDX48-apo (PDB: 2HXY; pink) and DDX3X-apo (PDB: 5E7I; green).

(C) Comparison of the RNA binding pocket between DDX21-AMPPNP-ssRNA (magenta) and Vasa-AMPPNP-ssRNA (PDB: 2DB3; blue), DDX19-AMPPNP-ssRNA (PDB: 3G0H; cyan) and DDX48-AMPPNP-ssRNA (PDB: 2HYI; yellow). ssRNA in DDX21-AMPPNP-ssRNA structure was colored in green, and AMPPNP was colored as a yellow stick. The 'DF loop', 'TG loop', and motif V (and Va) in all four structures share high conservation. See also Figure 3E for details.

(D) Comparison of the RNA binding pocket between DDX21-apo structure (PDB: 6L5L; red) with structures of DDX48-apo (PDB: 2HXY; pink), DDX3X-apo (PDB: 5E7I; green). The ‘DF loop’ and ‘TG loop’ in all three structures share high conservation. See also Figure 3D and 3E for details.

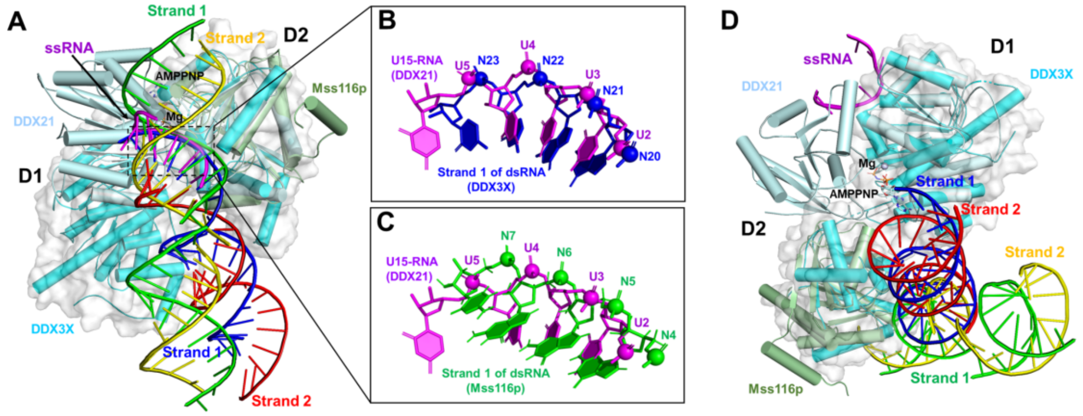

Figure S3. Superimposition of DDX21-AMPPNP-ssRNA with Mss116p-dsRNA and DDX3X-dsRNA structures

(A) Superimposition of DDX21-AMPPNP-ssRNA structure (PDB: 6L5N; palecyan) with DDX3X-dsRNA structure (PDB: 6O5F; protein in cyan and also outlined with a transparent molecular surface in white, and RNA strands in red and blue) and Mss116p-dsRNA structure (PDB: 4DB2; protein in palegreen and RNA strands in yellow and green). D1 is absent in the published Mss116p-dsRNA structure. The three structures are aligned based on the D2 domain.

(B) Superposition of U2-U5 in DDX21-AMPPNP-ssRNA structure (magenta) and N20-N23 of RNA strand 1 in the DDX3X-dsRNA structure (blue).

(C) Superposition of U2-U5 bound to D1D2 in DDX21-AMPPNP-ssRNA structure (magenta) and N4-N7 of RNA strand 1 in the Mss116p-dsRNA structure (green).

(D) Superimposition of DDX21-AMPPNP-ssRNA structure (PDB: 6L5N; palecyan) with DDX3X-dsRNA structure (PDB: 6O5F; protein in cyan and also outlined with a transparent molecular surface in white, and RNA strands in red and blue) and Mss116p-dsRNA structure (PDB: 4DB2; protein in palegreen and RNA strands in yellow and green). DDX21-AMPPNP-ssRNA structure was aligned with DDX3X-dsRNA structure based on the D1 domain, and Mss116p-dsRNA structure was aligned with DDX3X-dsRNA structure based on the D2 domain.

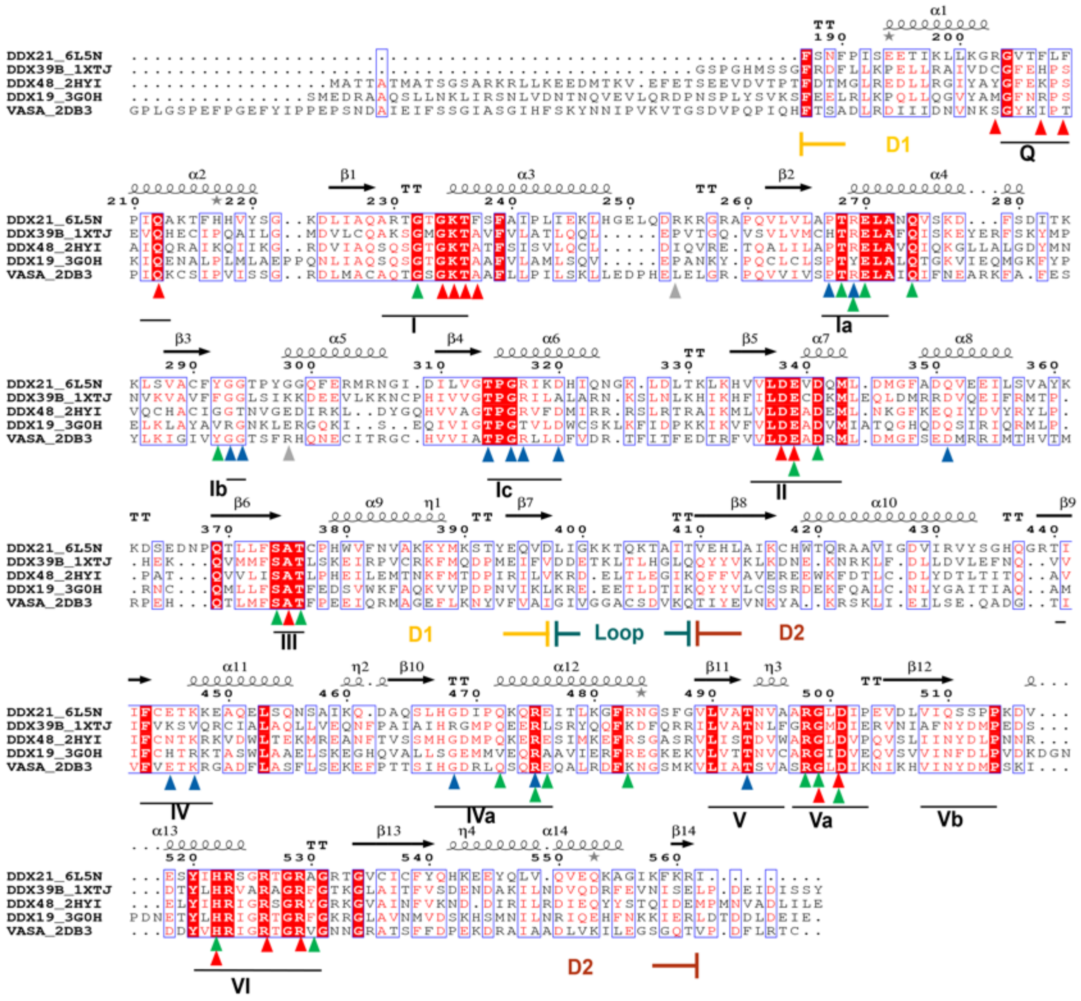

Figure S4. Structure-based sequence alignment of DDX21 with other DEAD-Box proteins

Red, blue, and green triangles indicate the residues involved in ATP binding, RNA binding, and inter-domain interactions, respectively.

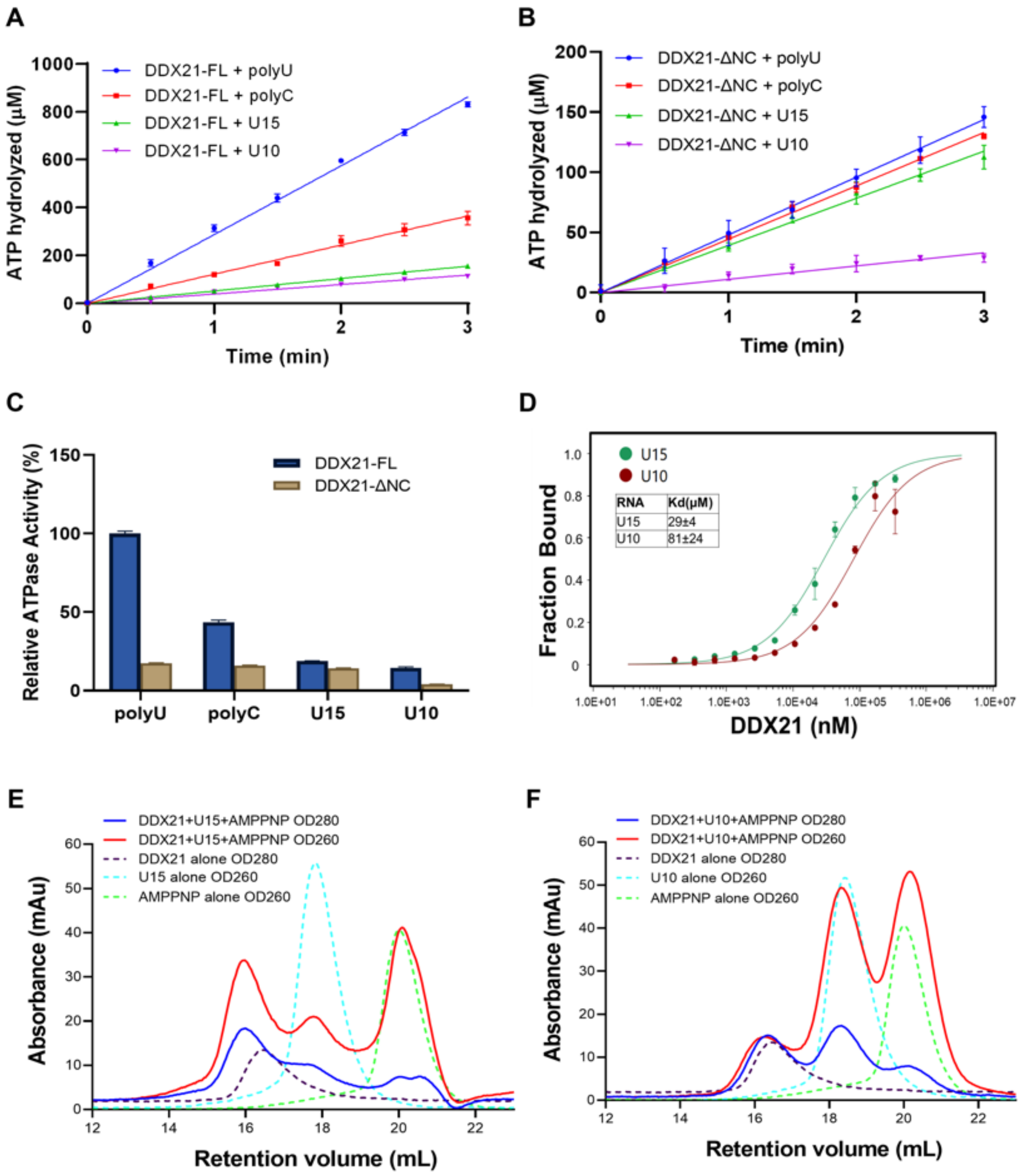

Figure S5. Comparison of ATPase activities stimulated by different polynucleotides

(A) ATPase activity of full-length DDX21 stimulated by poly(U), poly(C) and synthesized U15 or U10.

(B) ATPase activity of DDX21-ΔNC (188-563) stimulated by poly(U), poly(C) and synthesized U15 or U10.

(C) Histogram representation of ATPase activities shown in panel (A) and (B).

(D) The binding curves and Kd values for U15 or U10 with DDX21-ΔNC (188-563).

(E) Superimposition of the gel-filtration profiles among DDX21, U15, AMPPNP, and

the DDX21-U15-AMPPNP mixture. DDX21-U15-AMPPNP forms a stable complex in SEC.

(F) Superimposition of the gel-filtration profiles among DDX21, U10, AMPPNP, and the DDX21-U10-AMPPNP mixture. DDX21-AMPPNP and U10 could not form a stable complex in SEC.

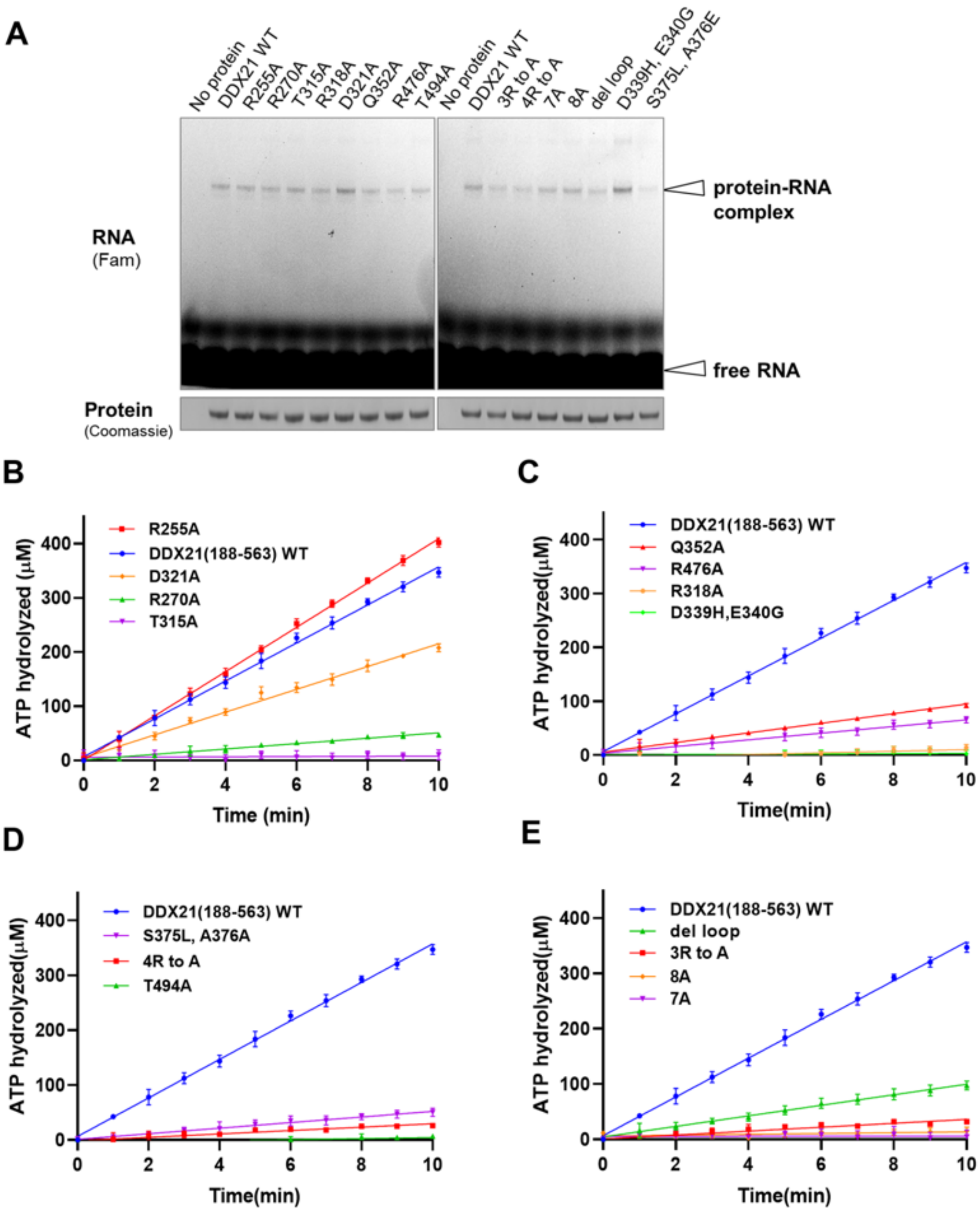

Figure S6. Mutational analysis with RNA-crosslinking and ATPase activity

(A) Crosslinking for Fam-labeled RNA (U15) with DDX21 (188-563) WT or mutants. The RNA-crosslinking assay was carried out by incubating 2 μM DDX21 and 2 μM 5'-Fam labeled 15-mer poly(U) RNA, in buffer containing 2 mM AMPPNP and 5 mM $MgCl_2$ at room temperature for 30 min, followed by irradiation with a 254 nm UV lamp for another 30 min, and then subjected to SDS-PAGE on a 4-12% polyacrylamide gel. (B) – (E) ATPase activities of DDX21 (188-563) WT and mutants. The OD360 readouts were converted to the concentrations of hydrolyzed ATP by a standard curve for inorganic phosphate that generated according to the kit manual.

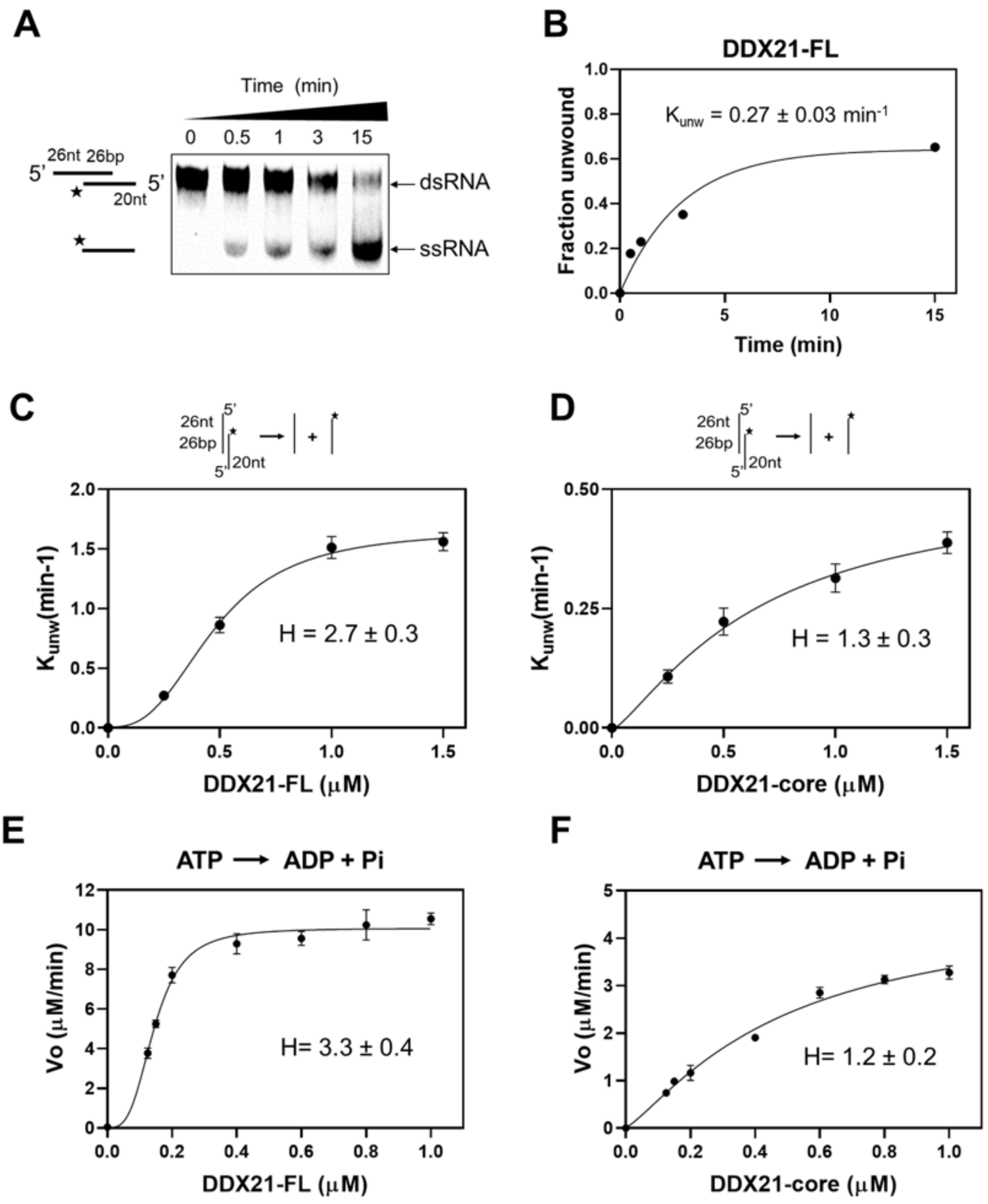

Figure S7. Hill cooperativity analysis of the ATPase and unwinding activities of human DDX21.

(A) Representative native PAGE image for a pre-steady unwinding reaction with 250 nM DDX21-FL and 10 nM 5'-tailed RNA substrate (26 bp dsRNA with 5' single-stranded overhangs), and 2 mM ATP/Mg^{2+}.

(B) Representative timecourse for the reaction shown in panel (A). Unwinding rate constant (K_{unw}) was determined from the curve fitting as described in the experimental section.

(C) Unwinding of the 5'-tailed RNA substrate (26 bp dsRNA with 5' single-stranded

overhangs; 10 nM) under pre-steady state conditions with increasing concentrations of full-length DDX21. Solid line is the best fit to the Hill equation (k_{unw}^{max} = 1.7 ± 0.1 min^{-1}, $K_{1/2}$ =480 ± 20 nM, H = 2.7 ± 0.3). Data points here and in all subsequent figures are averages from multiple independent measurements (N = 3) and error bars represent one standard deviation.

(D) Unwinding of the 5'-tailed RNA substrate (26 bp dsRNA with 5' single-stranded overhangs; 10 nM) under pre-steady state conditions with increasing concentrations of DDX21-core (188-563). Solid line is the best fit to the Hill equation (k_{unw}^{max} = 0.5 ± 0.1 min^{-1}, $K_{1/2}$ =660 ± 200 nM, H = 1.3 ± 0.3).

(E) RNA stimulated ATPase activity with 100 nM 5'-tailed dsRNA at indicated concentrations of full-length DDX21. Solid line is the best fits to the Hill equation (V_{max} = 10.1 ± 0.2 μM min^{-1}, $K_{1/2}$=150 ± 4 nM, H= 3.3 ± 0.4).

(F) RNA stimulated ATPase activity with 100 nM 5'-tailed dsRNA at indicated concentrations of DDX21-core (188-563). Solid line is the best fits to the Hill equation (V_{max} = 4.7 ± 0.6 μM min^{-1}, $K_{1/2}$=480 ± 110 nM, H= 1.2 ± 0.2).

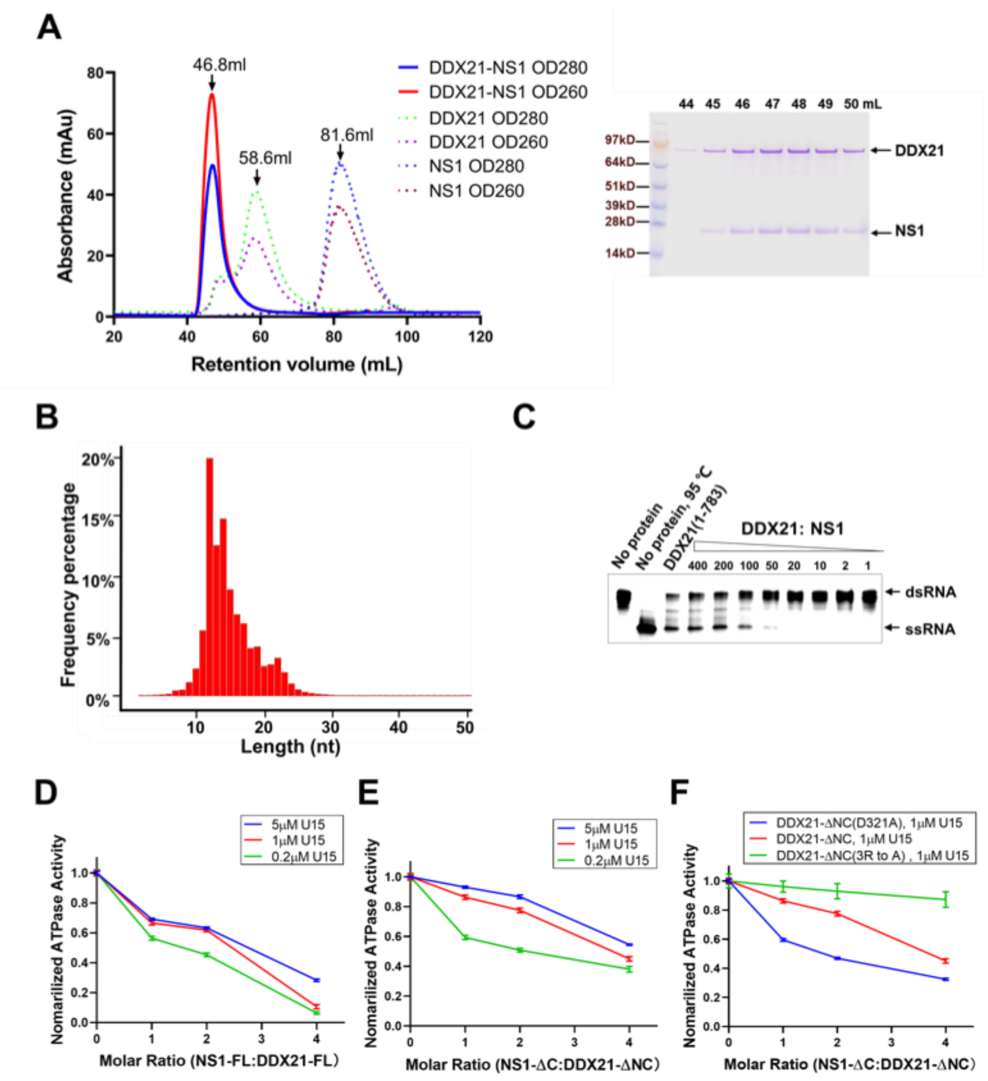

Figure S8. RNA modulates the interaction between DDX21 and NS1

(A) Gel filtration profile (left) and SDS-PAGE (right) of DDX21-NS1 complex that (A) was co-expressed (His-tagged full-length DDX21 and Flag-tagged full-length NS1) and co-purified from HEK293T cells by Ni-NTA column, followed with corresponding anti-flag column, and then loaded onto a Superdex200 10/300 column (GE healthcare, Boston, USA). The peaks with solid lines represent the purified complex and the peaks with dash lines represent purified DDX21 or NS1 alone as the controls.

(B) Distribution length statics of the extracted RNA from DDX21-NS1 complex, analyzed by Agilent 2100 Bioanalyzer.

(C) NS1 inhibits DDX21 ATP-dependent helicase activity. The ratio of RNA duplex with DDX21 was 1:10, and different molar ratios of full-length NS1 (1-237) were added with DDX21 for the unwinding inhibition assay. The final products of the reaction were

separated by a 4-20% gradient Novex™ TBE Gel (Thermo Fisher Scientific, Waltham, USA).

(E) – (F) NS1 inhibits DDX21 ATPase activity. The activities of full-length DDX21 with NS1 (C), DDX21-ΔNC (188-563) with NS1-ΔC (1-215) (D), and mutants of DDX21-ΔNC (188-563) with NS1-ΔC (1-215) (E) were detected in the presence of titrated U15. (C) and (D) contained concentration of 0.2 μM, 1 μM and 5 μM of U15, respectively, and (E) contained 1 μM of U15. The ratio of RNA duplex with DDX21 was 1:10.

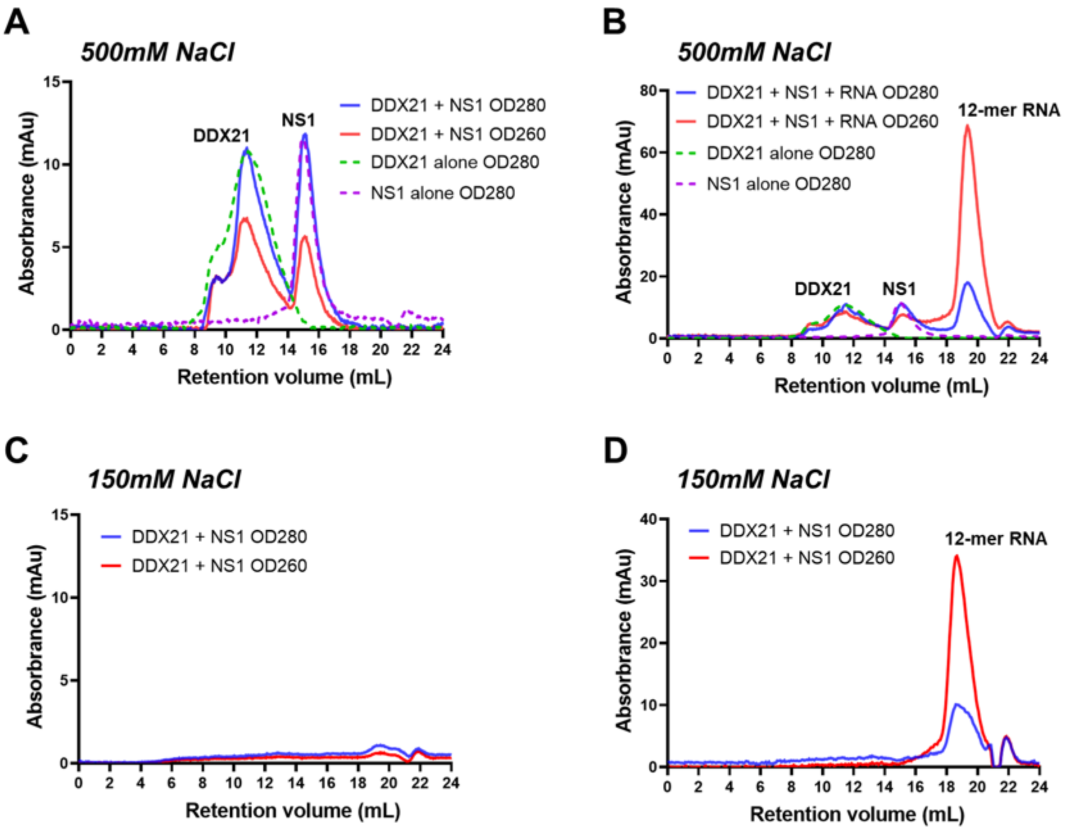

Figure S9. Size exclusion chromatography assays of DDX21 and NS1 with and without small RNAs

(B) Gel filtration co-elution profile for purified DDX21 and NS1 (solid line), by using a Superdex200 10/300 column (GE healthcare, Boston, USA). All subsequent figures were obtained from the same column. The peaks with dash lines represent purified DDX21 or NS1 alone as the controls. Full-length DDX21 and NS1 were purified with high salt concentration to remove bound nucleic acids, and mixed with 1:1 molar ratio for co-elution. They could not form a stable complex in the gel filtration with 500 mM NaCl.

(C) Gel filtration co-elution profile for purified DDX21, NS1 and the 12-mer small RNA from sequencing (see table S1), which were mixed with 1:1:2 molar ratios. The peaks with solid lines represent the mixture, and the peaks with dash line represent purified DDX21 or NS1 alone as the controls. They could not form a stable complex in the gel filtration with 500 mM NaCl.

(D) Gel filtration co-elution profile for purified DDX21 and NS1 (solid line). The purified full-length DDX21 and NS1 were not stable with 150 mM NaCl and no obvious peaks for them were observed.

(E) Gel filtration co-elution profile for purified DDX21, NS1 and the 12-mer small RNA from sequencing (see table S1), which were mixed with 1:1:2 molar ratios. The purified full-length DDX21 and NS1were not stable with 150 mM NaCl. No obvious peaks for them were observed, and only a peak of the small RNA was observed.

Table S1. Top 20 counts of RNAs with sequence information and frequencies from small RNA-sequencing

Sequence	Counts	Length(nt)
ACGGGAUUCUCU	470972	12
UCUGGUUGCUGAAC	141359	14
ACGGGAUUCUCC	69690	12
UAUCUGCGCUCUGC	60048	14
GAAGGUAACUGGCUUCAGC	53098	19
GGUGUAAUCGCGUGAAU	40060	17
UAUCUGCGCUCUGU	38228	14
UCUGCGCUCUGC	33683	12
ACGGGAUUCUCA	33555	12
CGAGCGAACGUG	33211	12
GCGUUGACUCUCAGU	33143	15
CGGUGCCUGACU	32279	12
GAAGGUAACUGGCUUCAGU	32262	19
UGGAUGCUGUACA	32133	13
UCUGGUUGCUGAAU	28277	14
UCAGCAACCAGAUC	27172	14
ACAUGGCACUCC	24975	12
AGAGAACCUUCG	24875	12
UAUACGGUCAACC	24554	13
GAAACGGUCUGAU	24478	13
